# Importance of Chemical Activation and the Effect of Low Operation Voltage on the Performance of Pt-Alloy Fuel Cell Electrocatalysts

**DOI:** 10.1021/acsaem.2c01359

**Published:** 2022-06-27

**Authors:** Matija Gatalo, Alejandro Martinez Bonastre, Léonard Jean Moriau, Harriet Burdett, Francisco Ruiz-Zepeda, Edwin Hughes, Adam Hodgkinson, Martin Šala, Luka Pavko, Marjan Bele, Nejc Hodnik, Jonathan Sharman, Miran Gaberšček

**Affiliations:** † Department of Materials Chemistry, National Institute of Chemistry, Hajdrihova 19, 1000 Ljubljana, Slovenia; ‡ ReCatalyst d.o.o., Hajdrihova 19, 1000 Ljubljana, Slovenia; § Johnson Matthey Technology Centre, Blount’s Court, Sonning Common, Reading RG4 9NH, U.K.; ∥ Johnson Matthey Fuel Cells, Lydiard Fields, Great Western Way, Swindon SN5 8AT, U.K.; ⊥ Department of Analytical Chemistry, National Institute of Chemistry, Hajdrihova 19, 1000 Ljubljana, Slovenia; # University of Nova Gorica, 5000 Nova Gorica, Slovenia

**Keywords:** platinum-alloys (Pt−M), chemical activation, dealloying, proton exchange membrane fuel cells (PEMFCs), oxygen reduction reaction (ORR), lower voltage limit (LVL)

## Abstract

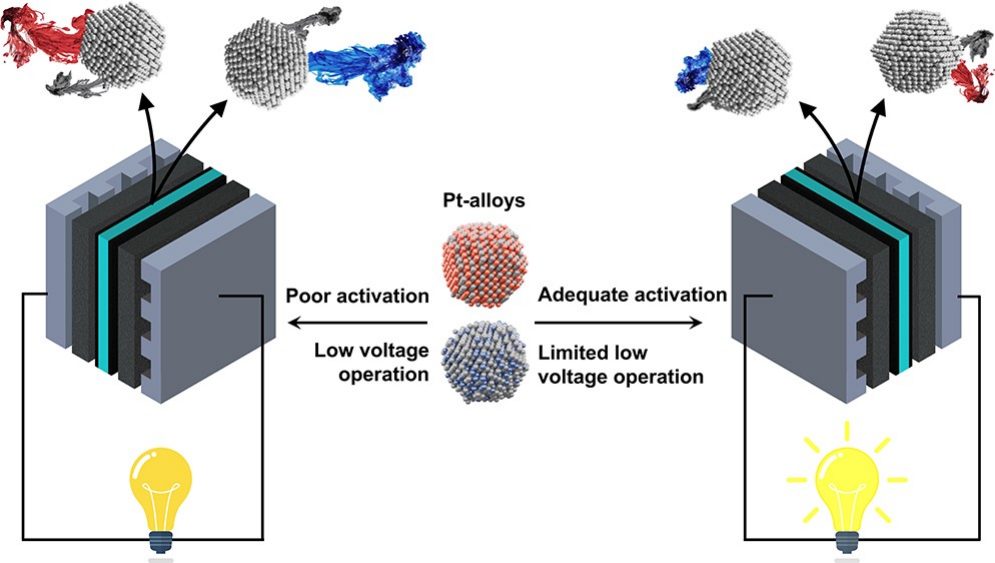

Pt-alloy (Pt–M) nanoparticles (NPs) with less-expensive 3d transition metals (M = Ni, Cu, Co) supported on high-surface-area carbon supports are currently the state-of-the-art (SoA) solution to reach the production phase in proton exchange membrane fuel cells (PEMFCs). However, while Pt–M electrocatalysts show promise in terms of increased activity for oxygen reduction reaction (ORR) and, thus, cost reductions from the significantly lower use of expensive and rare Pt, key challenges in terms of synthesis, activation, and stability remain to unlock their true potential. This work systematically tackles them with a combination of electrocatalyst synthesis and characterization methodologies including thin-film rotating disc electrodes (TF-RDEs), an electrochemical flow cell linked to an inductively coupled plasma mass spectrometer (EFC-ICP-MS), and testing in 50 cm^2^ membrane electrode assemblies (MEAs). In the first part of the present work, we highlight the crucial importance of the chemical activation (dealloying) step on the performance of Pt–M electrocatalysts in the MEA at high current densities (HCDs). In addition, we provide the scientific community with a preliminary and facile method of distinguishing between a “poorly” and “adequately” dealloyed (activated) Pt-alloy electrocatalyst using a much simpler and affordable TF-RDE methodology using the well-known CO-stripping process. Since the transition-metal cations can also be introduced in a PEMFC due to the degradation of the Pt–M NPs, the second part of the work focuses on presenting clear evidence on the direct impact of the lower voltage limit (LVL) on the stability of Pt–M electrocatalysts. The data suggests that in addition to intrinsic improvements in stability, significant improvements in the PEMFC lifetime can also be obtained *via* the correct MEA design and applied limits of operation, namely, restricting not just the upper but equally important also the lower operation voltage.

## Introduction

Electrification of the transport sector and the transition toward renewable energy solutions are crucial in the race toward cutting greenhouse gas emissions. While battery electric vehicles are already leading the way, an important part of the electrification puzzle will be fuel cell electric vehicles.^1^ One of the main barriers preventing mass adoption of the proton exchange membrane fuel cell (PEMFC) technology remains the high cost of the electrocatalyst material.^2^ Due to the inherently sluggish oxygen reduction reaction (ORR), a large amount of platinum (Pt) is required, which would represent over 50% of the total membrane electrode assembly (MEA) manufacturing costs,^3^ making it also the highest cost contributor to the entire stack.^4^ In addition, Pt is a low-earth-abundant, expensive critical raw material, which is limited in supply. To achieve significant cost reductions, the amount of Pt in the cathode electrocatalyst must be reduced. High activity, high electrochemically active surface area (ECSA), and high durability are all necessary for achieving significant improvements.^5,6^ While completely eliminating platinum group metals (PGMs) by the use of PGM-free electrocatalysts^7^ might seem a rational approach to reduce costs, achieving sufficient power densities as well as the durability of PGM-free electrocatalysts remains a major challenge, with currently no clear solution in sight.^6,8−10^ On the other hand, dealloyed Pt-alloy (Pt–M) nanoparticles (NPs) with less-expensive 3d transition metals (M = Ni, Cu, Co) supported on high-surface-area carbon supports are currently the state-of-the-art (SoA) solution to reach the production phase in PEMFCs^11,12^—resulting from their facile and already at scale preparation. Cost reduction using Pt-alloys is possible due to two key features: (i) Pt-alloys dilute Pt-atoms inside the nanoparticle’s core and thus improve Pt overall utilization;^13−16^ and (ii) they exhibit a higher kinetic activity toward the ORR due to a combination of a ligand, strain, coordination number, and/or surface disorder effects.^17−23^ This class of electrocatalysts has reached significant development as part of past Department of Energy (DoE) projects (in particular, dealloyed Pt–Co/C and Pt–Ni/C electrocatalysts)^24−26^ and resulted in record in-MEA activities that exceed the DoE performance targets^27^ of >0.44 A/mg_Pt_ with <40% activity loss after 30k voltage cycles in the rotating disc electrode (RDE) and MEAs.^28^ For this class of electrocatalysts, alloying is usually obtained *via* high-temperature thermal annealing.^29^

In addition to the importance of the bulk crystal structure of the Pt-alloy NPs, the dealloying (activation) step of the electrocatalyst also holds crucial importance.^30,31^ Relatively recently, transition-metal cation (*e.g.*, Ni, Co, Cu, Fe, etc.) contamination in the PEMFC has been recognized as another probable major contributor to the poor high current density (HCD) performance when using Pt-alloys.^32−35^ On the other hand, certain transition-metal cations were shown to diffuse into the membrane, causing formation of hydrogen peroxide radicals (due to the Fenton reaction^36^) that attack the membrane, leading to failure of the PEMFC. Pt–Cu alloys have in the past shown severe durability issues due to proton starvation of the cathode as a result of Cu plating onto the anode Pt/C electrocatalyst and thus blocking the hydrogen oxidation reaction (HOR).^37,38^ Similar observations on the presence of Cu in the membrane and in the anode have been reported recently.^39^ One possible solution to mitigate this has been demonstrated by Mani and co-workers,^40^ where an already fabricated catalyst-coated membrane (CCM) was exposed to another round of acid washing. However, this solution is not suitable for any kind of mass production of MEAs. Thus, solutions are necessary at the level of catalyst production. Consequently, improved fundamental understanding of the influence of chemical activation (dealloying) on the electrochemical behavior of Pt-alloy electrocatalysts is critical.

In addition to the possible introduction of transition-metal cations in the MEA during the phases of electrocatalyst ink preparation and/or CCM fabrication, the leaching can also occur during the break-in procedures and/or because of the Pt–M electrocatalyst degradation that results in metal dissolution.^41^ However, on the topic of metal dissolution, not only the intrinsic properties of an electrocatalyst material but also the operational conditions of the PEMFC seem to play a crucial role. There is important evidence in the literature that highlight the significance of both the upper voltage limit (UVL) and the lower voltage limit (LVL) in limiting the degradation of Pt-based carbon-supported electrocatalysts in the PEMFC. For instance, Uchimura and co-workers^42^ showed that the ECSA loss increases when LVL is reduced from 0.8 to 0.6 V when performing accelerated degradation test (ADT) cycles while using a fixed UVL (0.95 V). At that time, the transient nature of Pt dissolution was not fully understood and, thus, the mechanistic interpretation of the increased degradation was not yet considered significant for Pt dissolution arising from the destabilization of the Pt-oxide species. This was changed with the introduction of coupling methodologies between electrochemical cells and an inductively coupled plasma mass spectrometer (ICP-MS) that enabled time-and-potential-resolved monitoring of dissolution of metals.^43−46^ The investigations revealed a significant link between Pt dissolution and cathodically reduced Pt-oxide resulting from the so-called oxide-place exchange mechanism.^43^ Lastly, Yoshida and co-workers^47^ affirmed already during the launch of the first-generation Toyota Mirai the importance of limiting both the UVL and LVL to increase the fuel cell lifetime. This was later confirmed during the investigation of the first-generation Toyota Mirai stack as part of the Department of Energy (DoE) Mirai Fuel Cell Vehicle report, revealing system-level limitations on the LVL of the stack.^48^ While high UVLs occurring during the start-up/shut-down of the PEMFC lead to severe carbon corrosion,^49^ the LVL seems to also play a significant role that is perhaps of particular importance for the successful implementation of Pt-alloys. For this reason, further fundamental insights are necessary to truly understand this phenomenon.

Thus, in the present work, particular attention was given to the effect of the dealloying (activation) step on the performance with TF-RDEs and in 50 cm^2^ MEAs in single cells under a range of conditions. Furthermore, an additional link is established that connects the dealloying step with the observed TF-RDE features, in-line metal dissolution trends (using an electrochemical flow cell (EFC) coupled to an inductively coupled plasma mass spectrometer (ICP-MS)), and corresponding single-cell performances. In addition, the effect of the LVL on the stability of Pt-alloys toward the dissolution of the less noble metal was investigated by a combination of both in-line metal dissolution measurements and accelerated degradation tests (ADT) in 50 cm^2^ single-cell data.

## Experimental Section

### Syntheses of d-Pt-Cu/C-NIC and d-Pt-Ni/C-NIC Electrocatalysts

The Pt–M (M = Ni or Cu) electrocatalysts were prepared in accordance with the processes already reported previously.^50,51^ Briefly, the electrocatalysts were prepared in three steps. In the first step, Pt NPs were deposited onto a commercial carbon black support (Ketjen Black EC300J) *via* the double passivation galvanic displacement method reported elsewhere.^50^ In the second step, the prepared composites with deposited Pt NPs were thermally annealed to obtain an alloy crystal phase. In the last step, the electrocatalysts were subjected to two different activation (acid washing) protocols, thus forming a Pt-rich overlayer *via* dealloying. Within this study, the Pt-rich overlayer should be understood that the topmost layers of the Pt-alloy NPs have a more Pt-rich chemical composition than the bulk of the Pt-alloy NPs. In other words, the Pt-rich overlayer does not indicate an ideal “core–shell” structure^52^ and still contains some of the less noble metal.^41^ The first protocol was performed in accordance with a work described previously.^30^ Briefly, the process involves four times washing in 1 M acetic acid under CO purge. These two electrocatalysts are denoted as d-Pt-Cu/C-NIC-A and d-Pt-Ni/C-NIC-A, and the activation protocol is denoted as A-activation. The second protocol involves 24 h washing in 0.5 M H_2_SO_4_ at 80 °C. This protocol was developed during the previous DoE projects.^24−26^ These two electrocatalysts are denoted as d-Pt-Cu/C-NIC-S and d-Pt-Ni/C-NIC-S, and the activation protocol is denoted as S-activation. The results within this study are then compared to the activated d-Pt-Ni/C-JM benchmark with a similar metal loading provided by Johnson Matthey (JM).

### X-ray Diffraction (XRD) Analysis

The powder X-ray diffraction (XRD) measurements of all samples were carried out on a PANalytical X’Pert PRO MPD diffractometer with Cu Kα1 radiation (λ = 1.5406 Å) in the 2θ range from 10 to 60° with the 0.034° step per 100 s using a full-opened X’Celerator detector. Samples were prepared on a zero-background Si holder.

### Transmission Electron Microscopy (TEM) Analysis (NIC)

Scanning transmission electron microscopy (STEM) imaging was carried out in a probe Cs-corrected scanning transmission electron microscope Jeol ARM 200 CF operated at 80 kV.

### Transmission Electron Microscopy (TEM) Analysis (JM)

The samples were examined in the JEM 2800 (scanning) transmission electron microscope using the following instrumental condition: voltage (kV) 200.

### Inductively Coupled Plasma-Optical Emission Spectrometry (ICP-OES) and Digestion

All reagents used were of analytical grade or better. For sample dilution and preparation of standards, ultrapure water (18.2 MΩ cm^–1^, Milli-Q, Millipore) and ultrapure acids (HNO_3_ and HCl, Merck-Suprapur) were used. Standards were prepared in-house by dilution of certified, traceable, inductively coupled plasma (ICP)-grade single-element standards (Merck CertiPUR). A Varian 715-ES ICP optical emission spectrometer was used. Prior to ICP-OES analysis, each electrocatalyst was weighted (approximately 10 mg) and digested using a microwave-assisted digestion system (Milestone, Ethos 1) in a solution of 6 mL of HCl and 2 mL of HNO_3_. Samples were then filtered, and the filter paper was again submitted to the same digestion protocol. These two times digested samples were cooled to RT and then diluted with 2% v/v HNO_3_ until the concentration was within the desired concentration range.

### Electrochemical Evaluation *via* a Thin-Film Rotating Disc Electrode (TF-RDE)

#### Preparation of Thin Films and the Setup

Electrochemical measurements were conducted with a CompactStat (Ivium Technologies) in a two-compartment electrochemical cell in a 0.1 M HClO_4_ (Merck, Suprapur, 70%, diluted by Milli-Q, 18.2 MΩ cm) electrolyte with a conventional three-electrode system. Ag|AgCl was used as a reference, and a graphite rod was used as a counter electrode. The working electrode was a glassy carbon disc embedded in Teflon (Pine Instruments) with a geometric surface area of 0.196 cm^2^. The Ag|AgCl reference was separated from both the working and counter electrodes with a salt bridge to avoid Cl^–^-ion contamination. Prior to each experiment, the two-compartment electrochemical cell was boiled in Milli-Q water for 1 h, and the electrode was polished to mirror finish with Al_2_O_3_ paste (particle size 0.05 μm, Buehler) on a polishing cloth (Buehler). After polishing, the electrodes were rinsed and ultrasonicated (Ultrasound bath Iskra Sonis 4) in a Milli-Q/isopropanol mixture for 5 min. Then, 20 μL of 1 mg mL^–1^ water-based well-dispersed electrocatalyst ink was pipetted on the glassy carbon electrode completely covering it and dried under ambient conditions. After the drop had dried, 5 μL of Nafion solution (ElectroChem, 5% aqueous solution) diluted in isopropanol (1:50) was added. The electrode was then mounted on the rotator (Pine Instruments). The Ag|AgCl reference electrode potential against the reversible hydrogen electrode (RHE) was determined before the start of the experiment.

#### Pt/C Reference

In the case of Pt/C benchmark (TKK, TEC10E50E-HT) electrocatalyst measurements, the electrode was placed in an Ar saturated electrolyte under potential control at 0.05 V_RHE_. The electrocatalyst was electrochemically activated (potential cycling activation (PCA)) for 200 cycles between 0.05 and 1.2 V_RHE_ with a scan rate of 300 mV s^–1^ at 600 rpm. After PCA, the electrolyte was exchanged with a fresh one and the electrode was no longer under any external potential control (*i.e.*, the conditions corresponded to the open-circuit potential—OCP). ORR polarization curves were measured in an oxygen saturated electrolyte at 1600 rpm in the potential window 0.05–1.0 V_RHE_ with a scan rate of 20 mV s^–1^. Ohmic resistance of the electrolyte was determined and compensated for as reported in ref (53). At the end of ORR polarization curve measurements, the electrolyte was purged with CO under the potentiostatic mode (0.05 V_RHE_) to ensure successful CO adsorption. Afterward, the electrolyte was saturated with Ar. CO-electrooxidation was performed using the same potential window and scan rate as in ORR, but without rotation and in an Ar saturated electrolyte. After subtraction of the background current due to capacitive currents, kinetic parameters were calculated at 0.9 V_RHE_ using the Koutecky–Levich equation.^54^ Electrochemically active surface area (ECSA_CO_) was determined by integrating the charge in CO-electrooxidation (“stripping”) experiments as described in ref (55). All potentials are given against the reversible hydrogen electrode (RHE), which was measured at the start of the experiment.

#### Pt-Alloy Electrocatalysts

The electrocatalysts were placed in the electrolyte without any potential control (at the OCP) in an oxygen saturated solution, and ORR polarization curves were measured immediately after measurement of ohmic resistance and its compensation (under the same conditions as in the case of the Pt/C benchmark electrocatalyst). The protocol for CO-electrooxidation following the ORR measurement was the same as described above. At the end of CO-electrooxidation, 50 cycles of PCA (0.05–1.2 V_RHE_, 300 mV s^–1^, 600 rpm) were performed under the same parameters as in the case of the Pt/C benchmark electrocatalyst. Likewise, the electrolyte was exchanged with a fresh one at the end. ORR polarization curves and CO-electrooxidation were measured once again under the same conditions as before PCA. Each Pt-alloy electrocatalyst was measured at least three times.

### Electrochemical Flow Cell Coupled to Inductively Coupled Plasma Mass Spectrometry (EFC-ICP-MS)

#### Electrochemical Flow Cell Setup

**Scheme 1:**
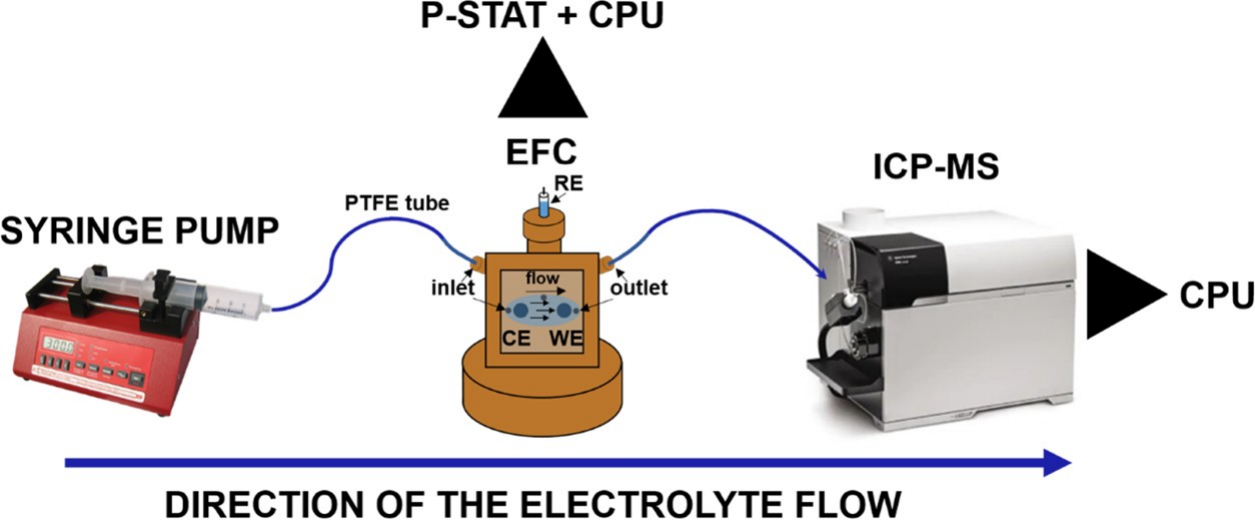
EFC-ICP-MS Setup Used for Obtaining Time-and-Potential-Resolved Metal Dissolution

The setup and measurement guidelines were established as part of previous works (Scheme 1).^30,31,41,44,56−59^ Briefly, the working and counter electrodes in the electrochemical flow cell (EFC) were glassy carbon discs (3 mm diameter) embedded into a polyether ether ketone (PEEK) material (BASi). The discs were aligned in series; the counter electrode was placed first and the working electrode second in the direction of the electrolyte flow. The sample was deposited on the electrode by drop-casting a 5 μL drop of the ultrasonically homogenized catalyst ink (1 mg mL^–1^). Such a preparation resulted in an electrocatalyst loading of 5 μg for all electrocatalysts. In addition, to increase the surface area of the counter electrode, a 5 μL drop of the Ketjen Black EC300J suspension (1 mg mL^–1^) was deposited on the glassy carbon counter electrode. After the drop had dried, 5 μL of Nafion solution (ElectroChem, 5% aqueous solution) diluted in isopropanol (1:50) was added. The Ag|AgCl reference electrode potential against RHE was determined before the start of the experiment. The housing of the cell was made from PEEK material, and the design was modeled after a commercial cross-flow cell (BASi, MF-1092, cross-flow cell). The volume of the cell was established with a home-made silicon gasket with 1.0 mm thickness and a 1.5 cm^2^ ellipsoidal cut. The carrier solution (0.1 M HClO_4_) was pumped through the cell at a constant flow of 400 μL min^–1^. Two glass syringes using Luer Lock connection to a poly(tetrafluoroethylene) (PTFE) tubing, two syringe pumps (WPI AL1000-220Z), and a diagonal four-way flow valve (Idex, V-100D) were used to enable a continuous flow of the solution.

#### ICP-MS

The EFC was coupled with an ICP-MS detector, namely, Agilent 7900ce ICP-MS instrument (Agilent Technologies, Palo Alto, CA), equipped with a MicroMist glass concentric nebulizer and a Peltier cooled Scott-type double-pass quartz spray chamber. A forward radio-frequency power of 1500 W was used with Ar gas flows: carrier 0.85 L min^–1^; makeup 0.28 L min^–1^; plasma 1 L min^–1^; and cooling 15 L min^–1^. The signals were recorded for Cu^63^, Ni^60^, and Pt^195^ with 0.5 s integration per data point. To convert the ICP-MS signals to concentration (ppb), standard solutions of Cu, Ni, and Pt in 0.1 M HClO_4_ were recorded with the following concentrations: 0.5, 1, 2, 5, 10, 20, 50, and 100 ppb.

#### Electrochemical Protocol

Electrochemical experiments were performed with a CompactStat (Ivium Technologies) with a typical three-electrode setup. No ohmic drop compensation method was used. The general electrochemical protocol is presented in Figure S1, Supporting Information (SI). Initially, Milli-Q water was pumped through the cell under open-circuit conditions (OCPs) before switching to 0.1 M HClO_4_. After 10 min of acid flow, the potentiodynamic protocol was started; to check for the effect of the lower potential limit (LPL), the electrocatalysts were cycled for three cycles between 0.925 and *X* V_RHE_ (*X* = 0.7, 0.65, and 0.6) with nine cycles in total (scan rate of 5 mV s^–1^). In another set of experiments, “memory effect” was checked by omitting *X* = 0.7 and 0.65 and only performing three cycles between 0.925 and 0.6 V_RHE_. In both cases, the experiment was followed with two cycles between 0.05 and 1.4 V_RHE_ (scan rate of 5 mV s^–1^). After each experiment, a sequence of potential pulses (see Figure S2, SI) was performed to synchronize the electrochemical experiment with the ICP-MS signal. Briefly, at the end of the experiment, the system is left at OCP for 200 s for the MS signal to reach background levels. This is followed by applying the first potential (oxidative) “pulse” (for 0.5 s) until 1.4 V_RHE_. After additional 150 s of waiting at OCP, a second potential (reductive) pulse (for 0.5 s) of 0.05 V_RHE_ is applied. Both pulses result in significant dissolution in the ICP-MS data and enabling one to observe the time difference between the electrochemical pulse and the detection of the metal dissolution on the MS.

### Single-Cell (MEA) Testing

#### MEA Fabrication

The MEAs used in this work consist of five layers. Nafion 1100EW (equivalent weight in g polymer/mol H+) was used to fabricate thin-layer electrodes. The cathode catalyst layers were prepared at an ionomer/carbon weight ratio of *ca.* 0.9 and metal loadings of *ca.* 0.10 mg_Pt_ cm^–2^, unless specified otherwise. The anode catalyst layer was kept constant at an ionomer/carbon weight ratio of *ca.* 1.5/1 and a metal loading of 0.1 mg_Pt_ cm^–2^. The membrane used was a perfluorosulfonic acid type, fabricated at JMFC with a thickness of *ca.* 20 μm. Catalyst layers were produced on a PTFE substrate and transferred *via* a decal method onto the membrane. Single cells (50 cm^2^ active area) were assembled by sandwiching the catalyst-coated membranes between the gas diffusion layers (GDLs) and applying an average compression onto the active area.

#### Fuel Cell Testing

The fuel cell station was built in-house at JMFC. Pure oxygen and synthetic air were used as cathode reactants and pure H_2_ as the anode reactant (all gases of 99.9% purity). Stoichiometric flow rates of anode (*s* = 2) and cathode (*s* = 9.5 for O_2_ and *s* = 2 for air) reactants were used at current densities >0.2 A cm^–2^ and constant flows (corresponding to 0.2 A cm^–2^ flows) at <0.2 A cm^–2^. Reactant humidification was achieved by water-bubblers, the temperatures of which were calibrated to yield the desired relative humidity (RH) values. Humidity and cell pressure were measured at the inlet for both electrodes. Cell resistances as a function of current density (*i.e.*, the sum of the proton-conduction resistance in the membrane and the various electronic resistances, bulk, and contact resistances) were determined using Hioki at 1 kHz. Multiple-path serpentine flow-fields (two and three parallel channels for the anode and cathode, respectively) machined into sealed graphite blocks were used for testing.

The MEAs were conditioned by the application of a constant current density of 500 mA cm^–2^ under H_2_/Air at 50 kPa gauge, 100% RH, and 80 °C. The cell voltage was monitored until a stable value was observed. The conditioning step lasted 2 h unless specified otherwise. Afterward, the cathode catalyst layer was exposed to a series of cathode starvation steps (see below) followed by a 2 h current hold at 500 mA cm^–2^ until a stable voltage was observed. After the starvation steps, the MEA was ready for testing by a series of H_2_/O_2_ polarization curves for mass activity (MA) quantification at different stages of the protocol (50 kPa gauge, 100% RH, and 80 °C). The polarization curves were recorded from low (*i.e.*, 0.05 A cm^–2^) to high current (*i.e.*, 2 A cm^–2^) ascending direction and backward, descending direction. The current density was maintained for 3 min at each step, and the MA value was obtained from the ascending polarization curve at 0.9 V by extrapolation resistance correction. H_2_-crossover current densities were measured using the procedure described by Kocha et al.^60^ In this test, the hydrogen that permeates through the membrane to the cathode is oxidized by the application of a voltage (typically 250–300 mV is sufficient, and the last one above 400 mV is in the mass transport limit), and the resulting current was measured. Therefore, the cell was operated under H_2_/N_2_, and the gas crossover measurements were done at each of the operating conditions (*i.e.*, temperature and H_2_-partial pressure). The catalyst activities were evaluated based on H_2_-crossover corrected current densities, *i*_eff_ (*i.e.*, *i*_eff_ = *i* + *ix*, with *ix* being on the order of 2–5 A cm^–2^). MA values reported in this article were not corrected for H_2_-crossover because the measured crossover currents accounted for a maximum of 10% at the loadings used in this study.

The ECSA was measured with the CO-stripping method using the cell in the half-cell mode where the anode electrode acts as a pseudo-reference electrode. The cathode voltage was controlled at 0.125 V at 80 °C, 100% RH, and 50 kPa gauge while purging with 1% CO in N_2_ at 300 mL min^–1^ for 15 min. Afterward, the cathode was purged with N_2_ at the same flow rate for 2 h to ensure that CO is removed from the bubblers and the catalyst layer pores. The adsorbed CO is oxidized electrochemically by scanning the cathode voltage from 0.125 to 0.85 V and back to 0.05 V, at 20 mV s^–1^ for three cycles. The area under the CO oxidation peak is integrated by subtracting the third scan from the first scan and using a 420 μC cm^–2^ constant for a CO monolayer on Pt.

For the voltage window experiments, the benchmark d-Pt-Ni/C-JM cathode catalyst was tested for durability under H_2_/N_2_ with the upper potential limit (UPL) fixed at 0.925 V, while the LVL was changed to 0.5, 0.6, or 0.7 V. At both LVL and UVL, the voltage was held for 3 s, while the rise time in between the voltage window of (LVL–0.925) V was below 0.5 s. The durability protocol was applied for a total of 1000 cycles, at 80 °C, 100% RH, and ambient pressure at the cell outlet. Afterward, the cell voltage was measured at 1.2 A cm^–2^ at different temperatures as described in the text.

## Results and Discussion

**Figure 1:**
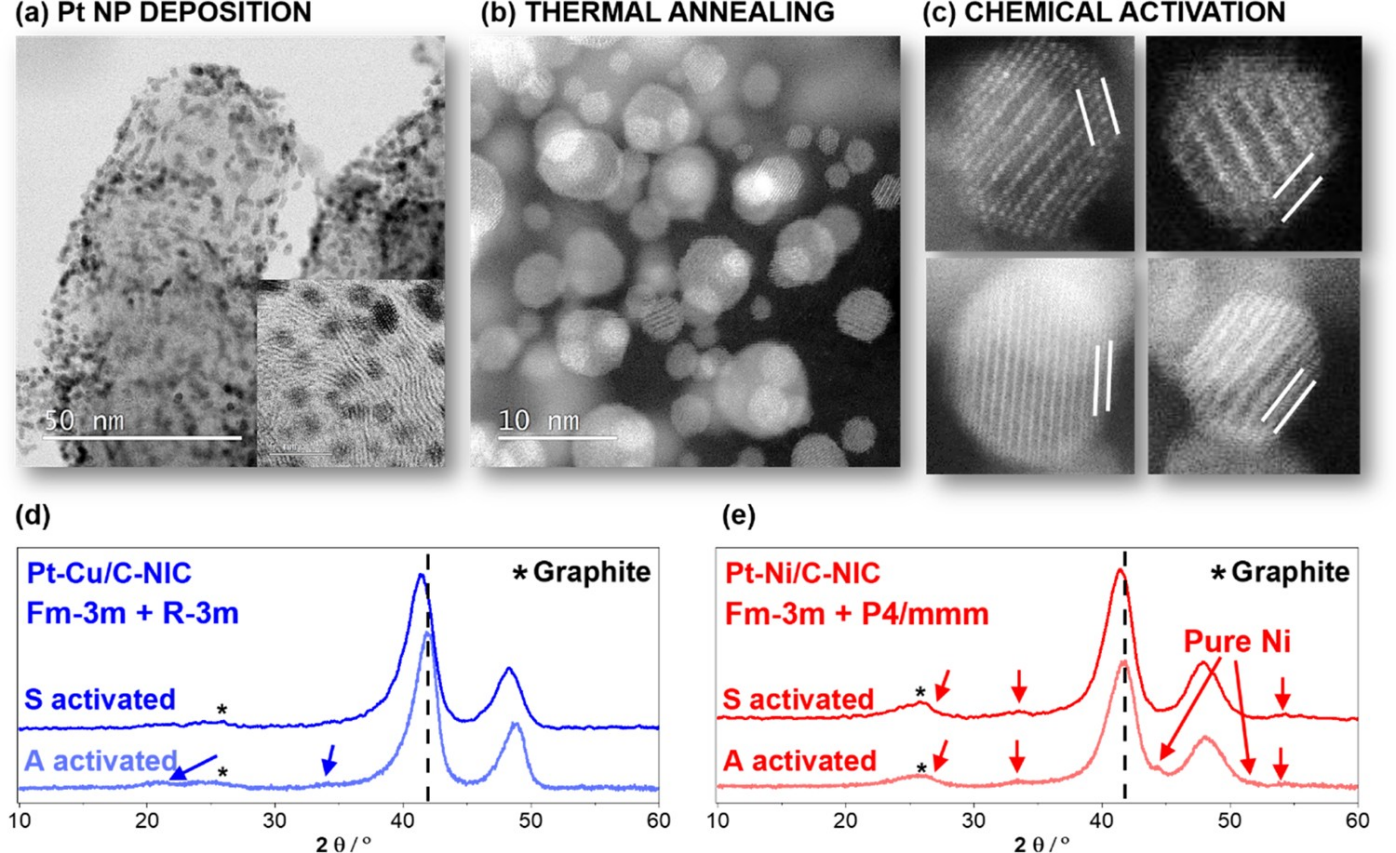
Three-step process used for preparation of Pt–M NIC electrocatalysts that involves (a) Pt NP deposition, (b) thermal annealing, and (c) chemical activation (white lines indicate the Pt-rich overlayer). XRD analyses of (d) d-Pt-Cu/C-NIC (A-activated and S-activated) and (e) d-Pt-Ni/C-NIC (A-activated and S-activated) electrocatalysts. See also Figures S3–S5 for additional TEM characterization. Arrows indicate the presence of a small fraction of the intermetallic phase or the pure M phase.

Figure 1a–c provides an overview of the three main synthesis steps of the Pt-alloy NIC electrocatalysts. In the first step (Figure 1a), Pt-based NPs were deposited on the M/C composites using our previously reported proprietary double passivation method.^50,51^ In the second step (Figure 1b), the obtained Pt-containing composites were subjected to a high-temperature thermal annealing to form a Pt-alloy structure.^29^ In the last step (Figure 1c), for the purpose of this study, both Pt–M alloys (M = Cu or Ni) were dealloyed using two different chemical activation (in other words, acid washing) protocols. In one case, we have employed a rather mild previously reported dealloying protocol (4 × 1 M acetic acid + CO_g_^30^) that uses mild conditions such as acetic acid, but in combination with an adsorptive gas such as CO_g_. The adsorptive gas binds to the Pt-surface and inhibits any possible readsorption of redeposition of M ions (M = Cu, Ni, or possibly other less noble metals) onto the Pt-surface. This improves the ability to wash the dealloyed M out of the carbon matrix during the process of acid washing.^30^ Hereinafter, we will refer to this acid-washing activation as protocol “A” and the electrocatalysts d-Pt-Ni/C-NIC-A and d-Pt-Cu/C-NIC-A. On the other hand, the second dealloying activation protocol included the use of a stronger acid such as sulfuric acid (H_2_SO_4_), also reported by others as part of previous DoE projects (0.5 M H_2_SO_4_, 24 h, 80 °C).^24−26^ Hereinafter, we will refer to this acid-washing activation as protocol “S” and the electrocatalysts d-Pt-Ni/C-NIC-S and d-Pt-Cu/C-NIC-S. Furthermore, the results within this study are compared to the d-Pt-Ni/C-JM benchmark with a similar metal loading provided by Johnson Matthey (JM).

STEM observations of both dealloyed Pt-alloy electrocatalysts (see Figures S3–S5, SI) provide evidence of a very high loading and uniform density of NPs over the carbon support. Additionally, a closer inspection reveals the detailed structure of several NPs, which include a Pt-rich overlayer (Figure 1c; see also Figures S3–S5, SI). In addition, the observed width of the main X-ray diffraction (XRD) peaks (Figure 1d,e) correlates well with the TEM characterization. Furthermore, whereas the majority of the observed Pt–M NPs in the present samples contain most likely the (disordered) *Fm*3*®m* crystal phase, a small fraction of the intermetallic (ordered) phase is also observed with both TEM and XRD. In the case of the “A-activated” Pt–Cu catalyst (Figure 1d), a small amount of *R*3*®m* (rhombohedral) characteristic of the 1:1 (Pt–Cu) ratio is observed.^61^ On the other hand, the A-activated Pt–Ni catalyst (Figure 1e) exhibits a small fraction of the tetragonal intermetallic crystal structure that is characteristic for the 1:1 (Pt–Ni) ratio.^62^ In addition, it exhibits some leftover pure-Ni phase that is still present even after the mild A-activation protocol. In contrast, the A-activated Pt–Cu catalyst does not exhibit any pure Cu phase. Upon the exposure of both catalysts to the harsher S-activation protocol, we see two distinct changes in the case of the S-activated Pt–Cu catalyst (Figure 1e). First, the position of the most intense peaks at approximately 42 and 49° experienced a significant shift toward lower angles, which correspond to a substantially more Pt-rich crystal structure. Second, any small fraction of the previously present superlattice peaks that correspond to the intermetallic phase disappeared into the background. This means that while the goal of the dealloying was to form a Pt-rich overlayer, the rather strong S-activation also affected the bulk properties of the intermetallic Pt–Cu core and partly disordered the crystal structure to the point where the intermetallic phase is no longer visible under the used measurement conditions due to the too small domain size.^30,63^ On the other hand, the exposure of the Pt–Ni electrocatalyst to the harsher S-activation (Figure 1e) also resulted in the successful removal of the pure-Ni phase. However, in contrast to the more M-rich Pt–Cu catalyst, the stronger S-activation did not significantly change the bulk composition of the Pt–Ni catalyst, resulting in only a very slight shift in the position of the most intensive XRD peaks. In addition, unlike in the case of Pt–Cu (Figure 1d), the presence of a small fraction of the intermetallic phase for the Pt–Ni catalyst remained unchanged even after the harsher S-activation (Figure 1e).

Table 1 provides a comparison of the liquid half-cell (thin-film rotating disc electrode; TF-RDE) performance of the electrocatalysts used in this study. For all of the electrocatalysts, the electrochemically active surface area was obtained by the integration of the CO-electrooxidation (ECSA_CO_) peak, while the kinetic performance for ORR was measured at both 0.9 and 0.95 V_RHE_ (specific activity, SA, and mass activity, MA).^55^ The liquid half-cell TF-RDE performance was evaluated to confirm the eligibility of the Pt–M NIC electrocatalysts for further investigation in 50 cm^2^ single cells. This required the catalysts to exhibit a superior kinetic ORR performance in contrast to SoA Pt/C electrocatalysts^64^ as well as an ECSA_CO_ higher than at least 40 m^2^ g_Pt_^–1^.^5^ According to expectations, the liquid half-cell TF-RDE kinetic performance of all Pt–M NIC catalysts at both 0.9 and 0.95 V_RHE_ exceeds that of Pt/C from Tanaka Kikinzoku Kogyo (TEC10E50E-HT; see Figure S6 for TEM characterization and Figure S7 for the ORR polarization curve and CO-electrooxidation). In addition, the measured ORR performances are comparable to or even exceed that of the d-Pt-Ni/C-JM benchmark. Lastly, evaluation of ECSA_CO_ for all Pt–M NIC electrocatalysts also confirmed the suitability for further evaluation in single-cell measurements at JM testing facilities.

**Table 1:** Comparison of ECSA_CO_, SA, and MA in Liquid Half-Cell (TF-RDE)^a^

electrocatalyst	ECSA_CO_ (m^2^ g_Pt_^–1^)	SA@0.9 V (mA cm^–2^)	MA@0.9 V (A mg_Pt_^–1^)	SA@0.95 V (mA cm^–2^)	MA@0.95 V (A mg_Pt_^–1^)
Pt/C reference (TEC10E50E-HT)	53.4	0.4	0.23	0.06	0.03
d-Pt-Ni/C-JM	65 ± 1	1.92 ± 0.21	1.25 ± 0.13	0.21 ± 0.02	0.14 ± 0.01
d-Pt-Ni/C-NIC-A	70 ± 2	1.46 ± 0.01	1.03 ± 0.02	0.20 ± 0.02	0.14 ± 0.01
d-Pt-Ni/C-NIC-S	76 ± 4	1.30 ± 0.08	0.99 ± 0.11	0.15 ± 0.01	0.12 ± 0.01
d-Pt-Cu/C-NIC-A	77 ± 2	1.94 ± 0.09	1.52 ± 0.10	0.28 ± 0.02	0.22 ± 0.02
d-Pt-Cu/C-NIC-S	77 ± 2	1.92 ± 0.03	1.48 ± 0.15	0.27 ± 0.03	0.23 ± 0.06

a See also Table S1 for metal contents and other parameters.

**Figure 2:**
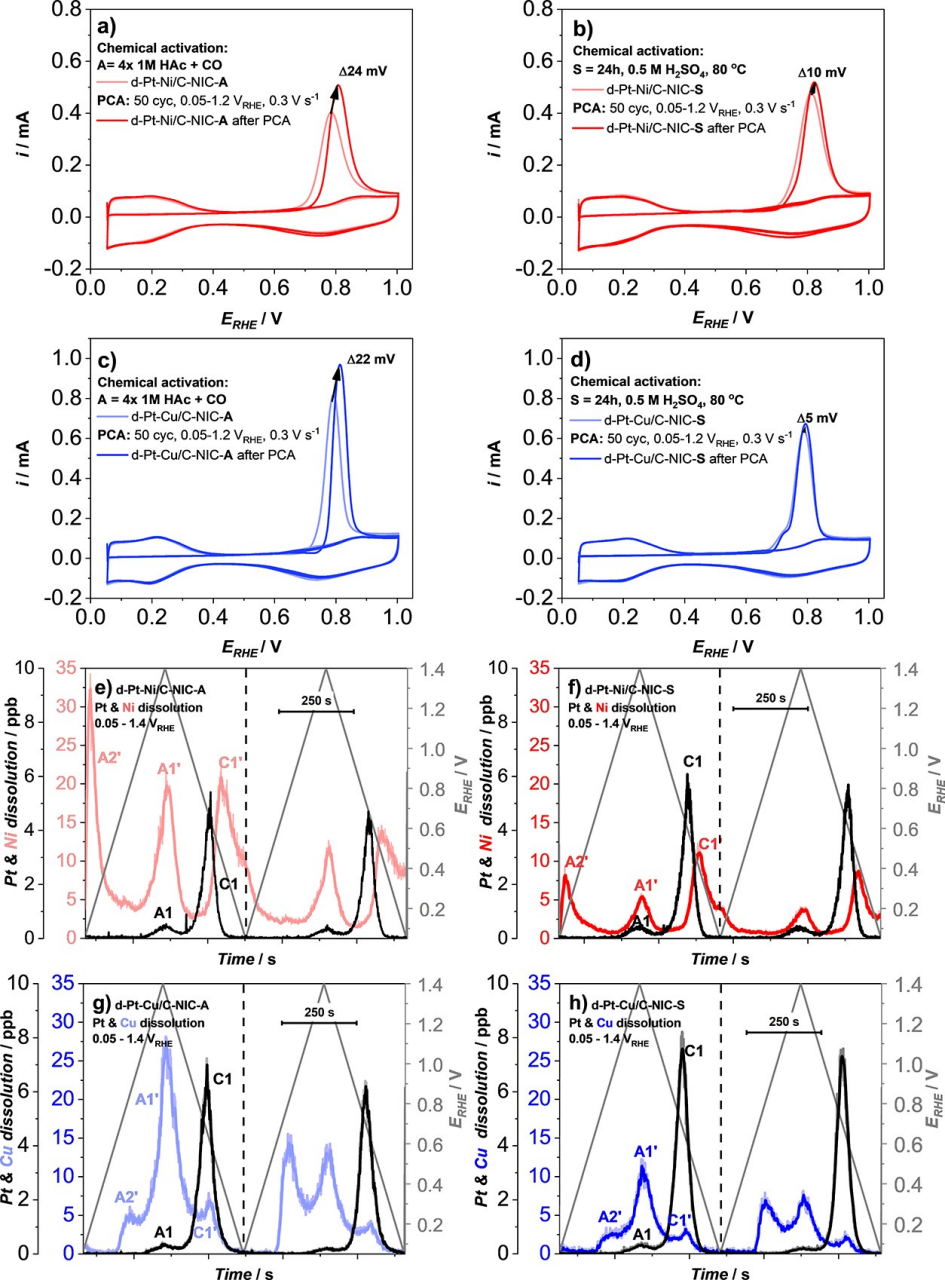
CO-electrooxidation comparison measured in the liquid electrolyte with TF-RDE for (a, c) d-Pt–M/C-NIC-A (M = Ni or Cu) and (b, d) d-Pt–M/C-NIC-S (M = Ni or Cu) before and after an additional 50 cycles of PCA (0.1 M HClO_4_, 0.05–1.2 V_RHE_, 300 mV s^–1^, 600 rpm rotation during activation and exchange of electrolyte prior to and at the end). Pt and M (M = Ni or Cu) dissolution measured with EFC-ICP-MS in a flow of 0.1 M HClO_4_ (wide potential window (WPW); two cycles 0.05–1.4 V_RHE_, 5 mV s^–1^) for (e) d-Pt-Ni/C-NIC-A, (f) d-Pt-Ni/C-NIC-S, (g) d-Pt-Cu/C-NIC-A, and (h) d-Pt-Cu/C-NIC-S.

Prior to the single-cell evaluation, a more in-depth analysis was conducted to provide a baseline understanding of the differences between A- and S-activation protocols on both the liquid half-cell TF-RDE electrochemical properties as well as in-line metal dissolution (Figure 2; see also Figures S8–S10, SI). In the case of TF-RDE measurements, both A- and S-activated catalysts were first evaluated by measuring the ORR (0.1 M HClO_4_, 1600 rpm, O_2_ saturated, *iR* corrected and background corrected, 20 mV s^–1^) followed by CO-electrooxidation (0.1 M HClO_4_, no rotation, Ar saturated, 20 mV s^–1^). This was then followed by an additional 50 electrochemical cycles of potential cycling activation (PCA; 0.05–1.2 V_RHE_, 300 mV s^–1^, 0.1 M HClO_4_). After PCA, ORR and CO-electrooxidation were measured under the same conditions once again.

Figure 2a shows that in the case of d-Pt-Ni/C-NIC-A, upon additional dealloying initiated *via* 50 cycles of PCA (0.05–1.2 V_RHE_, 300 mV s^–1^), the peak maximum corresponding to CO-electrooxidation at approximately 0.8 V_RHE_ shifts toward a more positive potential for ∼24 mV. However, for the case of more harshly activated d-Pt-Ni/C-NIC-S (Figure 2b), this potential shift reduces to only 10 mV. Despite the observed shift in CO-electrooxidation, there is interestingly no effect on the ORR polarization curves corresponding to their kinetic performances measured in half-cell TF-RDE before and after PCA for both A- and S-activated catalysts, respectively (see Figure S8a,b, SI). In addition, when comparing the 2nd (start) and 50th (end) cyclovoltammograms (CVs) of the PCA, a slight change in the distinct features corresponding to the Pt-surface such as the hydrogen underpotential deposition (H_UPD_) region and Pt-oxide formation and reduction regions can be observed (see Figure S8c,d, SI). When comparing the changes in these features for both A- and S-activated catalysts, respectively, unlike with the difference in the CO-electrooxidation maximum shift, no major difference distinguishing both activation protocols is revealed. Interestingly, the exact same 10 mV difference in the CO-electrooxidation peak maximum before and after PCA was observed in the case of the d-Pt-Ni/C-JM benchmark (see Figure S9a, SI). However, similar to both A- and S-activated Pt–Ni catalysts, PCA again had no effect on the ORR polarization curves corresponding to their kinetic performances measured in half-cell TF-RDE before and after PCA (see Figure S9b, SI). In addition, the changes in Pt-surface features when comparing the 2nd (start) and 50th (end) CVs of the PCA are also similar (see Figure S9c, SI).

To confirm that the observed effects are not a feature of the Pt–Ni alloy system, the exact same TF-RDE protocol was also performed on the d-Pt-Cu/C-NIC-A and d-Pt-Cu/C-NIC-S electrocatalysts (Figure 2c,d). Intriguingly, the exact same trends are observed even in the case of a completely different Pt-alloy system. Once again, d-Pt-Cu/C-NIC-A experiences a significantly larger potential shift in the CO-electrooxidation peak maximum measured before and after PCA (shift = 22 mV; Figure 2c) when compared to the S-activated catalyst (shift = 5 mV; Figure 2d). Furthermore, similar to the Pt–Ni catalysts, the ORR polarization curve comparison corresponding to the kinetic performances before and after PCA again reveals no visible difference for both A- and S-activated catalysts, respectively (see Figure S10a,b, SI). Lastly, also the comparison of distinct features corresponding to the Pt-surface in the 2nd and 50th CVs of the PCA (see Figure S10c,d, SI) reveals only slight changes for both A- and S-activated Pt–Cu catalysts, respectively. The take-away message here is that following our TF-RDE protocol, only a slight difference in the shift of the CO-electrooxidation peak maximum provides a possible clue that the A- and S-activated catalysts could in fact be quite different despite exhibiting a similar ORR activity in TF-RDE.

We can get the first confirmation of these differences by measuring the respective intrinsic metal dissolution profiles of activated Pt-alloy electrocatalysts using a well-established^41,44,56−59,65^ highly sensitive method, namely, electrochemical flow cell (EFC) coupled to an inductively coupled plasma mass spectrometer (ICP-MS). This advanced electrochemical characterization method allows us to track on-line time-and-potential-resolved dissolution of metals (Figure 2e–g; see also Figure S9d, SI). Other variations of the methodology used by other research groups, instead of the EFC, include usage of a scanning flow cell (SFC)^45,66,67^ or a variation used in combination with an RDE system.^68,69^ The intrinsic metal dissolution was monitored in accordance with the protocol presented in Figure S1. Briefly, each Pt-alloy electrocatalyst was exposed to the open-circuit potential conditions (I. OCP). This was followed by evaluation of the lower potential limit (II. LPL) effect (three cycles each of LPL, 0.925–0.*X* V_RHE_; *X* = 70/65/60, 5 mV s^–1^, 0.1 M HClO_4_) that will be presented in detail later on. Lastly, the LPL protocol was followed by two more wide potential window (III. WPW) cycles between 0.05 V_RHE_ and the upper potential limit (UPL) of 1.4 V_RHE_ (also 5 mV s^–1^) to initiate substantial dissolution of Pt and, consequently, also dissolution of the less noble metal. In other words, we wanted to probe how much of the less noble metal gets dissolved if we severely damage the “stable” Pt-rich surface. If parts II. and III. would be reversed, the WPW cycles (0.05–1.4 V_RHE_) could already substantially deplete the intrinsically unstable less noble metal from the Pt-rich overlayer and highly affect the results of the LPL studies presented in the next chapter. This is because the quantities of metal dissolution are substantially higher when Pt-based electrocatalysts are exposed to a wider potential window.^31,67^

As observed in the case of WPW cycles (0.05–1.4 V_RHE_, 5 mV s^–1^, 0.1 M HClO_4_; Figure 2e–g; see also Figure S9d, SI), one can expect two typical transient Pt dissolution peaks.^43−45,57,70−72^ Usually, this includes a smaller peak corresponding to the anodic (A1) and a more dominant cathodic (C1) Pt dissolution. The anodic (A1) Pt dissolution mechanism involves surface structure roughening caused by the oxide-place exchange mechanism.^43^ This creates Pt defects (low coordination sites), which do not get passivated by oxide formation and are thus prone to dissolution.^45^ On the other hand, with further penetration of oxygen in the crystal lattice of the NPs, reduction of this Pt-oxide results in formation of a much larger amount of unstable Pt defects, leading to a significant cathodic (C1) dissolution.^43^ Furthermore, what seems to be a general feature for all Pt-alloys^41^ is that following both the A1 and C1 Pt dissolution, we also observe A1′ and C1′ less noble metal dissolution. In other words, every time Pt dissolves, this exposes previously protected M atoms and causes their subsequent dissolution.^31^ In addition to that, Pt-alloys usually also exhibit another peak related to the less noble metal dissolution (A2′). However, what is special in this case is that A2′ does not have a corresponding Pt dissolution peak such as A1′ and C1′. For instance, the A2′ Cu dissolution peak (Pt–Cu alloy; Figure 2h,g) is related to the desorption (stripping) of Cu from the Pt-surface (Cu_UPD_).^30,31,37,38,41^ On the other hand, the origin of the A2′ Ni dissolution peak (Pt–Ni alloy; Figure 2e,f; see also Figure S9d, SI) cannot be yet determined accurately. What we can presume, however, is that in both cases, the alloyed less noble metal is stable until Pt-oxide (or perhaps the less noble metal oxide) is reduced.^73^ In accordance with the protocol presented in Figure S1 of the SI, after the end of II. LPL, the electrocatalyst is briefly exposed to OCP conditions, followed by a jump to 0.05 V_RHE_ (where the Pt-oxide gets reduced) and the start of WPW cycles. While A2′ Cu (Figure 2g,h) remains stable a bit further even in the metallic form due to its higher standard electrode potential, A2′ Ni (Figure 2e,f; see also Figure S9d, SI) starts to dissolve already at the beginning of the cycle (0.05 V_RHE_). Thus, we can claim that the A2′ dissolution definitely occurs on the Pt-alloy NPs. A last but also important difference to note in relation to the WPW cycles (0.05–1.4 V_RHE_, 5 mV s^–1^, 0.1 M HClO_4_; Figure 2f,g; see also Figure S9d, SI) is comparing the differences in A1′ and C1′ dissolution intensities. While both d-Pt-Ni/C-NIC-S (Figure 2f) and the d-Pt-Ni/C-JM benchmark (see Figure S9d, SI) exhibit a higher intensity of C1′ Ni dissolution than A1′, the situation is reversed in the case of d-Pt-Cu/C-NIC-S (Figure 2g). Partly, this can be explained by the fact that during the cathodic scan, part of the dissolved Cu deposited back to the Pt-surface as Cu_UPD_ and only gets stripped away during the next anodic scan, resulting in an increased A2′ Cu dissolution peak in the next cycle (Figure 2g,h).^30,31,41^ Nevertheless, the A1′ Cu dissolution in the case of the Pt–Cu alloy is still significantly higher than that of the Pt–Ni alloy, which is in accordance with our prior works.^41,74^

However, the comparison of the total dissolved amounts of the less noble metal during the WPW cycles for d-Pt-Ni/C-NIC-A (Figure 2e) and d-Pt-Ni/C-NIC-S catalysts (Figure 2f) reveals a significantly different amount of Ni dissolution out of Pt–Ni NPs. While the amount of dissolved Pt within each cycle is comparable for both catalysts (due to similar Pt loadings on the glassy carbon electrodes), the more harshly S-activated catalyst loses substantially less Ni within each WPW cycle. Analogously to the same CO-electrooxidation peak maximum shift behavior of the d-Pt-Ni/C-JM benchmark, the Ni dissolution once again resembles more the behavior of the S-activated Pt–Ni NIC catalyst (see Figure S9d, SI). A slight difference most likely originates due to the slightly higher metal (Pt and Ni wt %) loadings in the case of d-Pt-Ni/C-NIC-S (see Table S1, SI). Analogously, also comparison of d-Pt-Cu/C-NIC-A (Figure 2g) and d-Pt-Cu/C-NIC-S catalysts (Figure 2h) reveals a significantly higher amount of Cu dissolution out of Pt–Cu NPs in the case of the A-activated catalyst.

Because single-cell testing is time consuming, requires relatively large amounts of the electrocatalyst (in contrast to TF-RDE), and is highly complex in nature (many parameters can influence performance), it is highly important to be able to have a preliminary and facile method of distinguishing between a “poorly” or “adequately” dealloyed (activated) Pt-alloy electrocatalyst. Providing such a solution has been one of the core goals of this study. Namely, the data so far suggests that both A-activated analogues not only exhibited a larger shift in the potential of the CO-electrooxidation peak maximum (Figure 2a–d) but also experienced a higher amount of less noble metal dissolution (Figure 2e–h). Thus, based on the evidence presented in this study (as well as in our previous reports^30,31^), we argue that the potential difference in the CO-electroxidation peak maximum can serve as a very simple and sensitive preliminary indicator that can help distinguish between a poorly or adequately dealloyed (activated) Pt-alloy electrocatalyst. The effect of the less noble metal ions on the position of the CO-electrooxidation peak has already been explored on the Pt/C electrocatalyst by Durst and co-workers.^75^ In their study, they spiked the electrolyte with different amounts of various metal ions and observed shifts in the position of the CO-electrooxidation peak toward more negative potentials. This occurs because less noble metal ions induce a more facile (hydr)oxide formation at the Pt-surface in the double layer. Hydrated less noble metal cations are located between the inner and outer Helmholtz planes where they partially lose their hydration shell and come closer to the Pt-surface, inducing an increase in the OH_ad_ coverage that can cause the shift in the onset of CO-electrooxidation toward lower potentials.^75^ Following this study, we explored this on Pt-alloys as part of our previous work where also a more “mild” electrochemical activation (potential hold activation) resulted in a shift of the CO-electrooxidation peak maximum toward lower potentials in comparison to a harsher one (potential cycling activation).^30,31^ However, the present study, for the first time, provides the necessary data that correlates this effect with “real” electrocatalyst samples based on the used chemical activation protocol (A- or S-activation). Thus, if our assumptions are correct, a similar correlation should be observed when performances of A- and S-activated Pt–M NIC analogues are evaluated in the 50 cm^2^ single cells.

The comparison of kinetic performances (SA and MA at 0.9 V) and ECSA_CO_ (at 100% RH) for all investigated electrocatalysts in a 50 cm^2^ single cell (MEA) is presented in Table 2. Under this study, all electrocatalyst powders were treated under identical conditions during ink formulation (I/C ratio of 0.9) and all catalyst layers were printed and treated identically. As a result, it was observed that the Pt cathode loading on A-activated analogues was higher than on S-activated analogues. This is most likely due to different presences of transition-metal cations in the catalyst inks that affected the ink viscosity. However, the mass activity results reported have been adjusted for the difference in Pt loading. First and foremost, it is interesting that despite d-Pt-Cu/C-NIC-A being the most active electrocatalyst according to TF-RDE characterization (Table 1), it has the lowest MA among all of the investigated electrocatalysts. This already suggests that the presence of Cu ions in a single cell is significantly more damaging than Ni (similar to Co^38^). On the other hand, the kinetic performance of d-Pt-Ni/C-NIC-A is also worse than that of the S-activated catalyst despite the trend being reversed in TF-RDE (Table 1). In addition, there is also a slight increase in ECSA_CO_ as a result of the dissolved pure-Ni phase upon exposure of the electrocatalyst to the S-activation protocol (Figure 1e). This trend is in good agreement with the ECSA evaluation by TF-RDE (Table 1). In contrast to A-activated catalysts, results of both S-activated Pt-alloy catalysts provide a similar kinetic performance at low current densities against the d-Pt-Ni/C-JM benchmark. Furthermore, with the exception of d-Pt-Cu/C-NIC-A, the trends are in good agreement with the TF-RDE data (Table 1), where kinetic performances of S-activated electrocatalysts were also comparable to that of the d-Pt-Ni/C-JM benchmark. However, although the kinetic performance that is comparable to the d-Pt-Ni/C-JM benchmark (Table 2) is already a good indication, it by far does not reveal the entire story.

**Table 2:** Comparison of Kinetic Performance at 0.9 V, ECSA_CO_, and Average High-Frequency Resistances (HFRs) in 50 cm^2^ Single Cells of A- and S-Activated Pt–Ni and Pt–Cu NIC Catalysts as well as the Benchmark d-Pt-Ni/C-JM

						HFR (mΩ cm^–2^)	
electrocatalyst	Pt loading (μg_Pt_ cm^–2^)	I/C ratio	SA@0.9 V (mA cm^–2^)	MA@0.9 V (A mg_Pt_^–1^)	ECSA_CO_ (m^2^ g_Pt_^–1^)	100% RH	30% RH
d-Pt-Ni/C-JM	99.6	0.9	1.00	0.53	55.3	48	61
d-Pt-Ni/C-NIC-A	139	0.9	0.872	0.49	57	64	85
d-Pt-Ni/C-NIC-S	81.7	0.9	1.07	0.51	68.7	52	65
d-Pt-Cu/C-NIC-A	162	0.9	0.611	0.34	56	66	85
d-Pt-Cu/C-NIC-S	88	0.9	0.903	0.56	64.3	56	76

**Figure 3:**
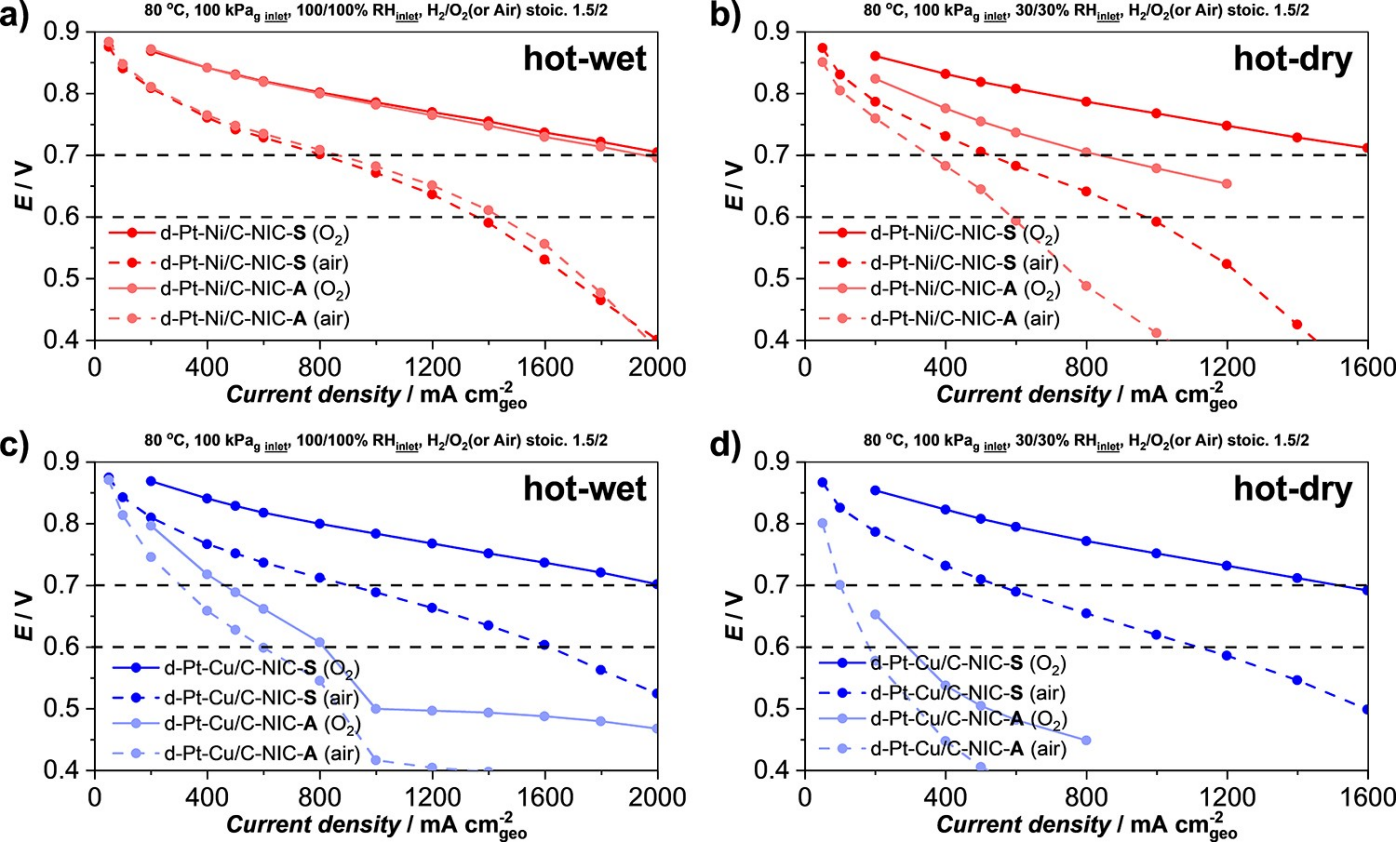
Comparison of ORR polarization curves measured in 50 cm^2^ single cells under H_2_/O_2_ and H_2_/Air (anode/cathode) using the Pt-alloy cathode catalysts shown in Table 2. General test conditions are specified in the figure. (a) Comparison of A- and S-activated Pt–Ni catalysts at hot-wet conditions (80 °C, 100% RH), (b) A- and S-activated Pt–Ni catalysts at hot-dry conditions (80 °C, 30% RH), (c) A- and S-activated Pt–Cu catalysts at hot-wet conditions (80 °C, 100% RH), and (d) comparison of A- and S-activated Pt–Cu catalysts at hot-dry conditions (80 °C, 30% RH). Single-cell ORR polarization data on the d-Pt-Ni/C-JM electrocatalyst can be found in Figure S11, SI.

To understand the entire story, we need to look at the 50 cm^2^ single-cell ORR polarization curves and, thus, HCD performances at various conditions (Figure 3). Namely, both A- and S-activated catalysts were compared in both O_2_ and air, as well as at both “hot-wet” (80 °C, 100% RH on both the cathode and the anode) and “hot-dry” (80 °C, 30% RH on both the cathode and the anode) conditions. Figure 3a shows the comparison of 50 cm^2^ single-cell ORR polarization curves between d-Pt-Ni/C-NIC-A and d-Pt-Ni/C-NIC-S electrocatalysts in hot-wet conditions in both O_2_ and air. Interestingly, no significant difference is observed in the O_2_ performance for both A- and S-activated catalysts up until 2 A cm_geo_^–2^, whereas impressively, the voltage did not yet drop below 0.7 V. In contrast, in accordance with expectations, performance of both A- and S-activated catalysts drops significantly when O_2_ on the cathode is exchanged for air. Nevertheless, both catalysts still achieve an impressive 1.4 A cm_geo_^–2^ at 0.6 V with the cathode loading of the S-activated catalyst being only ∼82 μg_Pt_ cm^–2^ (Table 2). Here, we wish to emphasize to the reader that no effort was put into catalyst ink/layer optimization in the case of Pt–M NIC electrocatalysts as part of this study, but rather, these parameters were translated based on previous works by JM.^34,76^ Another important note here is that the observed difference between both A- and S-activated Pt–Ni catalysts can be considered as an experimental error or a result of a slightly different Pt cathode loading (Table 2). Thus, these two performances can be considered very similar. However, despite the similar performance of both A- and S-activated Pt–Ni catalysts in hot-wet conditions, the same cannot be claimed when the hot-dry conditions are used (Figure 3b). Here, the d-Pt-Ni/C-NIC-S significantly outperforms d-Pt-Ni/C-NIC-A in both O_2_ and air. Looking now at both A- and S-activated Pt–Cu catalysts, the differences become even more significant. Already upon comparison of the performances in hot-wet conditions (Figure 3c), d-Pt-Cu/C-NIC-S significantly outperforms d-Pt-Cu/C-NIC-A. Interestingly, while the performance of approximately 2 A cm_geo_^–2^ at 0.7 V in O_2_ for the d-Pt-Cu/C-NIC-S is comparable to that of d-Pt-Ni/C-NIC-S, d-Pt-Cu/C-NIC-S achieves an impressive 1.6 A cm_geo_^–2^ at 0.6 V in air with a cathode loading of only 88 μg_Pt_ cm^–2^. When we move into the hot-dry conditions (Figure 3d), similar to both Pt–Ni catalysts, the performance of d-Pt-Cu/C-NIC-A is once again much worse than that of d-Pt-Cu/C-NIC-S. However, while the HCD performance (air curves) of d-Pt-Cu/C-NIC-S exceeded that of d-Pt-Ni/C-NIC-S under the hot-wet conditions (Figure 3a,c), the situation becomes reversed under the hot-dry conditions (Figure 3b,d).

This mainly suggests three things. (i) Comparing Pt-alloy cathodes at both wet and dry conditions can reveal performance differences related to the presence of the less noble metal ions in the MEA. In particular, the present data also suggests that measuring only at wet conditions could even be misleading and mask transport issues related to the presence of less noble metal ions. Thus, measuring at a lower (dryer) RH as well as in air rather than in O_2_ is critical for adequate evaluation of Pt-alloys, especially when considering and understanding the effects of the transition-metal cations on the performance. (ii) “Tolerance” for Ni ions in single cells far exceeds that of Cu ions. When comparing both A-activated Pt-alloy catalysts at hot-wet conditions (Figure 3a,c), d-Pt-Ni/C-NIC-A exhibits significantly better BoL performance than d-Pt-Cu/C-NIC-A despite having even a small amount of pure-Ni phase (Figure 1e) that most likely, in addition to leaching of Ni from Pt–Ni NPs, additionally contributed toward the additional contamination with Ni ions. However, transition-metal ions can also be introduced in the MEA upon Pt–M electrocatalyst degradation. Thus, the second part of this work focuses on the effect of the LVL on the dissolution of the less noble metal from the Pt–M electrocatalysts. (iii) As predicted by the prior observations in TF-RDE and EFC-ICP-MS (Figure 2), S-activated Pt–M NIC analogues significantly outperformed the A-activated ones in the 50 cm^2^ single cells, namely, at the HCDs. This not only supports our arguments on the CO-electrooxidation shifts but also suggests that the presence of the transition-metal cations in the CCM is one of the most critical parameters governing the performance of Pt-alloys in PEMFCs. However, as already suggested, even if the Pt-alloy is adequately activated and no significant amount of the less noble metal ions is introduced in the CCM at the BoL, due to aging of the Pt–M NPs, the operation of the PEMFC will over time introduce fresh transition-metal ions. Thus, in the second part of this study, the focus will be on understanding a highly underestimated parameter in PEMFC operation, the lower voltage limit (LVL).

The effect of the voltage window on Pt dissolution^43−45,57,70−72^ as well as on the dissolution of the less noble metal in the case of Pt-alloys^30,31,65^ has already been well documented. However, most of the existing studies focus on the understanding of the changes in UPL of 1.0 V or above (in other words, understanding what happens if Pt is more significantly oxidized). On the other hand, understanding the changes in LVL,^42^ especially at UVLs below 1.0 V, is perhaps similar to the electrocatalyst activation, vastly underestimated and understudied.^65,72^ Considering that the fuel cell stack will operate for the vast majority of its time within a voltage window of around 0.6–0.95 V, understanding the mechanism of Pt and Pt-alloy degradation in this regime is in fact critical.

**Figure 4:**
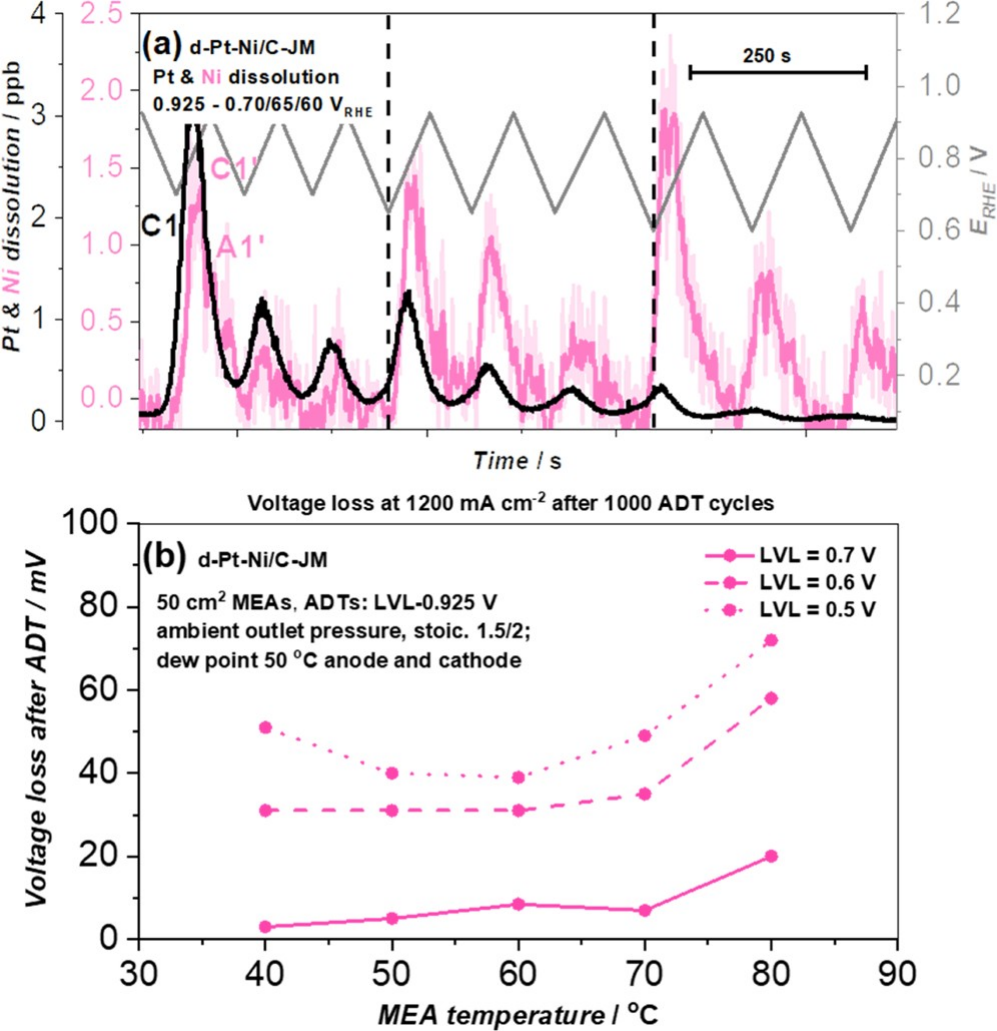
(a) Pt and Ni dissolution (LVL effect in the operational voltage window; 0.925–0.*X* V_RHE_; *X* = 0.70, 0.65, and 0.60; 5 mV s^–1^) for the d-Pt-JM/C-JM benchmark using EFC-ICP-MS. See also Figure S12 for reproducibility data and the memory-effect test. (b) Number of mV lost at 1200 mA cm_geo_^–2^ measured at different MEA temperatures after 1000 cycles of ADT using a UPL of 0.925 V and different LVL values, namely, 0.7, 0.6, and 0.5 V, upon using the d-Pt-Ni/C-JM benchmark as the cathode catalyst (as shown in Table 2).

Thus, we are hereby providing valuable evidence on the effect of the lower potential limit (LPL) on metal dissolution by initially using the EFC-ICP-MS (three cycles each LPL, 0.925–0.*X* V_RHE_; *X* = 70/65/60, 5 mV s^–1^, 0.1 M HClO_4_) and evaluating all five Pt-alloy electrocatalysts from this study. The experimental protocol consists of in total nine slow cycles with a scan rate of 5 mV s^–1^ in the operational potential window with a constant upper potential limit (UPL) and a decreasing LPL for 50 mV every three cycles. Figure 4a shows the effect of lowering the LPL from 0.7 to 0.65 V as well as from 0.65 to 0.6 V_RHE_ on the dissolution of both Pt and Ni in the case of the d-Pt-Ni/C-JM benchmark. Interestingly, while we have kept the UPL constant, lowering of the LPL results in an increase of Ni dissolution. Furthermore, we have tested this effect for reproducibility (see Figure S12a,b, SI) as well as excluded the possibility of a memory effect. Thus, in addition to the reproducibility measurements, where the initial LPL is 0.7 V_RHE_ (see Figure S12c,d, SI), we performed two more measurements where the LPL of 0.6 V_RHE_ was used right away (see Figure S12e,f, SI). This way, no prior metal transient dissolution and, thus, degradation took place prior to the three cycles with an LPL of 0.6 V_RHE_. By going directly to the lowest LPL of 0.6 V (see Figure S12e,f, SI), the observed Ni dissolution was indeed substantially higher than in the case with an LPL of 0.7 V_RHE_ (see Figure S12c,d, SI).

To provide further evidence on this important phenomenon, we extended our study by performing carefully designed accelerated degradation tests (ADTs) in 50 cm^2^ single cells at various LVLs and temperatures using the d-Pt-Ni/C-JM benchmark as the cathode electrocatalyst. Figure 4b shows the decrease in mV at 1.2 A cm^–2^, in 50 cm^2^ single cells, observed upon exposing the cathode catalyst layer to these ADTs (1000 cycles, 0.925–0.*X* V_RHE_; *X* = 70/60/50, 3 s hold at both LVL and UVL; ambient outlet pressures, stoichiometry 1.5/2, dew point 50 °C anode and cathode; H_2_/N_2_). The results clearly indicate that the highest voltage losses were observed in the case of ADT conditions with an LVL of 0.50 V_RHE_, whereas the voltage losses decrease dramatically if the LVL is increased to 0.60 or 0.70 V_RHE_. The trends presented in Figure 4b reveal another important parameter highly relevant for Pt-alloy cathodes. Regardless of the LVL used in the ADT (0.7, 0.6, or 0.5 V), the observed cell voltage losses after the ADT significantly increase with temperatures above 60 °C. This is, however, to be expected since temperature is also known to affect the kinetics of Pt-oxide formation and reduction, influencing the onsets.^71^ Consequently, at the same LVL, a higher fraction of Pt-oxide gets reduced with increasing temperature, leading to a lower protection of the less noble metal toward the dissolution and, thus, a higher voltage loss. The effects of both the voltage window and the temperature are in line with our recent liquid electrolyte follow-up study.^74^

As further evidence on the importance of the results in the present study, revealing data published by the Argonne National Laboratory^48^ with a detailed investigation of the operated Mirai stack showed that under system-controlled operation the voltage window was kept within the range 0.65–0.85 V. Analysis of MEAs showed no Co dissolution into the membrane under system-controlled operation, but that a large amount of Co had dissolved after the application of the ADT for catalyst durability defined by the US-DoE 0.60–0.95 V voltage window. The reported cathode loading for the Mirai MEAs under study was *ca.* 0.30 mg_Pt_ cm^–2^, and under the operating conditions used, this loading was enough to prevent exposure to aggressive upper and lower voltages (*i.e.*, <0.65 or >0.95 V). It is plausible that this benign voltage window leads to low Pt dissolution and is probably one of the causes that prevented significant dissolution of cobalt. Clearly, this provides solid evidence that the voltage window used to determine the durability of Pt-alloys is extremely important.

**Figure 5:**
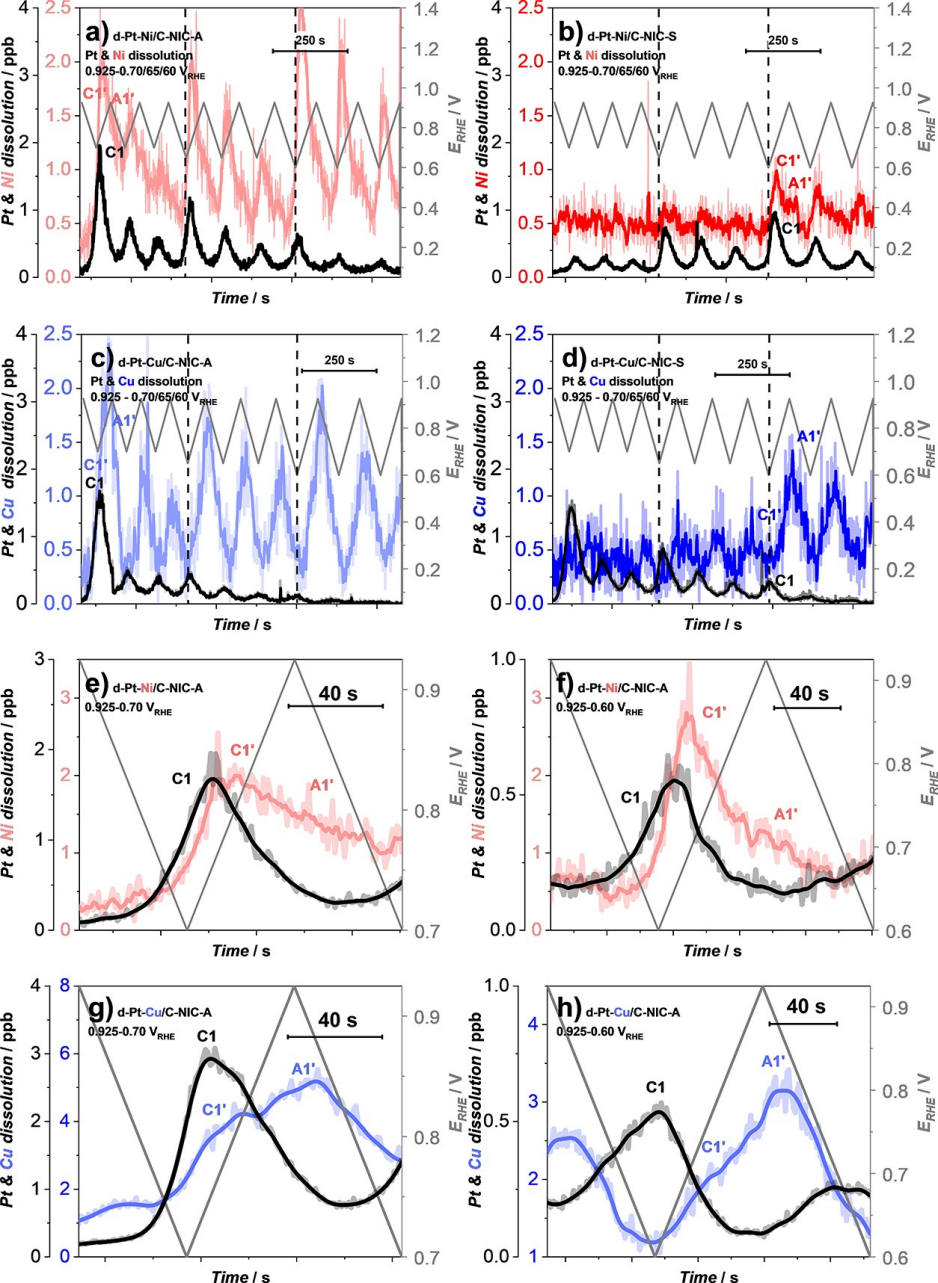
LPL effect (0.925–0.*X* V_RHE_; *X* = 0.70, 0.65, and 0.60; 5 mV s^–1^) comparison for A- and S-activated (a, b) Pt–Ni and (c, d) Pt–Cu catalysts using EFC-ICP-MS. Close-up metal dissolution profiles of the first and seventh cycles from five of the main manuscripts for both A-activated (e, f) Pt–Ni and (g, h) Pt–Cu alloy catalysts at LPLs of 0.7 and 0.6 V. Raw data in (a)–(h) have been smoothed for better visibility.

In addition to the d-Pt-Ni/C-JM benchmark, we have also tested all four A- and S-activated Pt-alloy catalysts (Figure 5a–d) using the exact same protocol presented in Figure 4a (see also Figure S1 for the II. LPL part of the EFC-ICP-MS protocol). First and foremost, we observe the exact same effect (in other words, increase the less noble metal dissolution upon the decrease of LPL) as in the case of the d-Pt-Ni/C-JM benchmark (Figure 4a). Also importantly, analogous to the comparison of metal dissolution in the WPW cycles for the A- and S-activated Pt-alloy electrocatalysts (Figure 2e–g), both A-activated catalysts exhibit a dramatically higher amount of less noble metal dissolution (Figure 5a,c) in contrast to the S-activated catalysts (Figure 5b,d). We wish to remind the reader that upon TF-RDE characterization (Figures 2a–d and S8–S10), the only noticeable difference between the A- and S-activated catalysts for both Pt–Ni and Pt–Cu catalysts was observed in the shift of the CO-electrooxidation peak maximum measured before and after PCA. However, following also the additional comparison of the metal dissolution trends (Figure 2) as well as single-cell performances (Figure 3), it is now rather clear that depending on the choice of the dealloying (activation) protocol, the electrochemical behavior is influenced substantially.

By focusing on only a single LPL cycle (Figure 5e–h) along with the mechanistic insights gained from the WPW cycles (Figure 2e–h), we can also start to make sense of the mechanisms behind the observed metal dissolution peaks. In both cases of Pt–Cu and Pt–Ni alloys (Figure 5e–h), we see a single peak corresponding to Pt dissolution. This peak corresponds to the reduction of Pt-oxide and, thus, cathodic transient dissolution of Pt (C1). Following the onset of the C1 Pt dissolution peak, we observe the onset of the peak corresponding to the dissolution of the less noble metal (C1′). Whereas we do not observe any A1 peak that would correspond to the anodic transient dissolution of Pt, a clear shoulder peak corresponding to the dissolution of the less noble metal A1′ is present for both Pt–Ni and Pt–Cu alloys. Since A1′ less noble metal dissolution can only be a direct consequence of the A1 Pt dissolution,^31,41^ it is highly likely that the A1 Pt dissolution is simply out of our limit of detection (in contrast to the significantly harsher WPW cycles; Figure 2e–h). Nevertheless, a careful inspection of the observed dissolution profiles reveals many other important messages. For instance, similar differences in the intensities of A1′ and C1′ less noble metal dissolutions between Pt–Ni (Figure 5e,f) and Pt–Cu (Figure 5g,h) can be observed as in the case of WPW cycles (Figure 2e,h). In other words, in the case of Pt–Ni alloy, C1′ Ni dissolution is dominant in comparison to A1′ Ni dissolution, whereas the situation is reversed in the case of Pt–Cu (with A1′ Cu dissolution being the dominant peak). This brings us back to the C1 Pt dissolution peak. Our evidence suggests that C1 cathodic Pt dissolution, as a consequence of oxide-place-exchange, is already the dominant Pt dissolution mechanism at a UPL of as low as 0.925 V (Figures 4a and 5). This goes in line with the observations made by Ahluwalia and co-workers where C1 Pt dissolution became dominant only above 0.9 V.^65^ Thus, at UPLs above 0.9 V, the choice of LPL governs the extent of the cathodic corrosion of Pt.^42^ Consequently, with more Pt dissolution, a higher amount of previously protected M gets exposed to the acidic environment, thus resulting in an increased dissolution of the less noble metal. This is in good agreement with the trends observed during ADT 50 cm^2^ single-cell tests (Figure 4b), where we can presume that the observed voltage losses at the HCDs can be correlated to the dissolution of Ni and its consequent interaction with the ionomer.^32,33^

As discussed in this paper, if the upper voltage is controlled at 0.925 V or lower, the LVL where almost full reduction of the Pt-oxide occurs,^45^ such as 0.60 V (*vs* RHE) used in this work, can be of particular importance to enable alloy cathodes. Of course, the voltage at which full Pt-oxide reduction occurs depends on the *T*, RH, and *p* used in addition to the composition of the cathode catalyst layer. The observations derived from this work lean toward the hypothesis that Pt-alloys are particularly prone to faster degradation compared to pure Pt if exposed to voltage windows where almost all of the Pt-oxide and OH_ad_ are removed and grown again using a dynamic profile, leading to the depletion of the less noble metal in the first three or four atomic layers and the presence of leached metal ions in the ionomer. The explanation is based on the hypothesis that oxygen diffusion into the subsurface is an important step in the formation of the surface oxide and for the subsequent Pt dissolution. For a detailed discussion on this topic, the reader is directed to the elegant work by Balbuena and co-workers^77^ on the mechanism of subsurface oxygen formation, surface segregation, and Pt dissolution for Pt and Pt-alloys. Considering that the less noble metal composition in the second and third atomic layers of a Pt-alloy is usually in the range of Pt/X (3:1) X = Ni, Co, or Cu, from the voltage windows provided in this work, one could easily explain the fast depletion of the less noble metal in the subsurface if exposed to the 0.6–0.925 V voltage window. Dissolution of the less noble metal is influenced by the reactions that occur in the vicinity of the Pt-oxide growth and reduction. On the other hand, if the catalyst surface is left slightly oxidized, such as with the use of narrower voltage windows 0.70–0.925 V, then the depletion of the less noble metal in the subsurface is inhibited under the protocol and conditions used in this work. One would also have to acknowledge that the onset of the oxide growth is shifted to higher voltages/potentials for the case of Pt-alloys compared to Pt.^78^

This is of particular relevance for industrial application in MEAs because by preventing the segregation of the less noble metal the negative impact of dissolved metal ions is avoided. This is often overlooked in wet electrochemical cells where dissolved metal ions in the electrolyte do not lead to a decrease in ORR activity. However, in MEAs, it is well known that metal ions can diffuse from the cathode layer to the membrane and back again due to the flux of ions back and forth from the cathode layer to the membrane, proportional to the flux of protons and hence the load. As a consequence, proton and oxygen transport can be severely decreased in the presence of dissolved metal ions, which are two of the main reasons for the decrease in performance at high current densities after cycling Pt-alloys, among other factors, as reported by Ramaswamy and co-workers.^79^

## Conclusions

In summary, to show the critical importance of the catalyst activation, we have prepared four dealloyed (chemically activated) Pt–M (M = Cu or Ni) electrocatalysts and compared them with the Pt–Ni alloy catalyst from Johnson Matthey. Namely, both Pt-alloys were subjected to either a “milder” acetic acid activation protocol or a “harsher” sulfuric acid activation protocol. Both A- and S-activated analogues were then evaluated using TF-RDE, in-line metal dissolution using the EFC-ICP-MS methodology, as well as tested in the 50 cm^2^ single cells at both the hot-wet (80 °C, 100% RH) and the hot-dry (80 ^o^C, 30% RH) conditions. In the present study, both A-activated analogues performed significantly worse in 50 cm^2^ MEAs, experienced a higher amount of less noble metal dissolution, and most importantly, exhibited a larger shift in the potential of the CO-electrooxidation peak maximum. Thus, a much simpler and more accessible technique like TF-RDE as a rather precise and very sensitive preliminary indicator (*via* CO-electrooxidation) can help distinguish between a poorly or adequately dealloyed (activated) Pt-alloy electrocatalyst. On the other hand, also following the protocols used in this study, one can gain insights into the chemical activation in relation to the less noble metal dissolution using the EFC-ICP-MS. Furthermore, evaluating Pt-alloy electrocatalysts in single cells at a lower RH (hot-dry) as well as in air rather than in O_2_ reveals significantly more information about the performance of Pt-alloy electrocatalyst performance than at high RH (hot-wet). Overall, it is clearly presented that the presence of the less noble metal ions in the CCM is one of the most critical parameters governing the performance of Pt-alloys.

In the second part of the study, the data generated using both EFC-ICP-MS and ADTs in 50 cm^2^ single cells provides clear evidence on the significant importance of the LVL/LPL in relation to the less noble metal dissolution. Thus, better understanding of Pt-oxide formation and reduction in relation to Pt dissolution as well as subsequent dissolution of the less noble metal is crucial to enable the use of Pt-alloy cathodes at HCDs needed for automotive conditions. We show that operating Pt-alloys below 0.7 V results in a significantly higher degree of less noble metal dissolution and, thus, higher voltage losses when using Pt-alloy cathodes in single cells even at relatively low UVLs/UPLs of 0.925 V. In addition, we discovered that oxide-place exchange responsible for Pt cathodic transient dissolution plays a crucial role in this. Being previously considered as highly important only at UPLs of above 1.1 V, our evidence suggests that it is in fact the dominant mechanism responsible for the observed performance losses already at operating voltages relevant for operation of Pt-alloys in PEMFCs. Since transient dissolution of the less noble metal is highly connected with the transient dissolution of Pt, following this logic, perhaps operational strategies at the system level^9,47^ as well as modifications inhibiting Pt dissolution and improving the protection of the less noble metal should be of a much larger focus in the future (by, for example, addition of Au^68,80−83^). Thus, in our opinion, there are still large opportunities not only in the investigation of novel chemical activation protocols but also in the development of highly stable high metal loaded/high ECSA Pt-alloy electrocatalysts and also in how system-level operation of Pt-alloy electrocatalysts should be viewed in contrast to traditional Pt/C for extending the long-term performance benefits of this still intriguing class of PEMFC electrocatalysts.

## Supporting Information Available

Electrochemical protocol used for evaluation of metal dissolution; electrochemical “pulses” methodology used for synchronization of the electrochemical and ICP-MS signals as exemplified on the d-Pt-Ni/C-NIC-S electrocatalyst; HAADF images; TF-RDE characterization in liquid electrolyte for the Pt/C reference; comparison of ORR polarization curves; liquid electrolyte TF-RDE comparison of CO-electrooxidation experiments with their respective follow-up cycles and ORR polarization curves for the SoA d-Pt-Ni/C-JM benchmark; comparison of ORR polarization curves; reproducibility and memory-effect evaluation of the LPL effect using the SoA d-Pt-Ni/C-JM benchmark using EFC-ICP-MS in the flow of 0.1 M HClO_4_; additional data on the electrocatalysts used in this study such as wt % of Pt and M, mass of Pt and electrocatalyst used for the liquid electrolyte TF-RDE measurements; and corresponding area of the CO-electrooxidation peak (PDF)
