# Supplementary material for: Importance of Chemical Activation and the Effect of Low Operation Voltage on the Performance of Pt-Alloy Fuel Cell Electrocatalysts
Source: ACS Appl Energy Mater. 2022 Jun 27;5(7):8862–77. doi: 10.1021/acsaem.2c01359 (PMC9326812; doi:10.1021/acsaem.2c01359)
Supplement: Supplementary file 1 — ae2c01359_si_001.pdf [file ae2c01359_si_001.pdf]

Supporting information: The Importance of Chemical Activation and the Effect of Low Operation Voltage on the Performance of Pt-alloy Fuel Cell Electrocatalysts

Matija Gatalo[a,b], Alejandro Martinez Bonastre[c,], Léonard Jean Moriau[a], Harriet Burdett[c], Francisco Ruiz-Zepeda[a], Edwin Hughes[c], Adam Hodgkinson[d], Martin Šala[e], Luka Pavko[a], Marjan Bele[a], Nejc Hodnik[a,f*], Jonathan Sharman[c], Miran Gaberšček[a]*

[a] Department of Materials Chemistry, National Institute of Chemistry, Hajdrihova 19, 1000 Ljubljana, Slovenia

[b] ReCatalyst d.o.o., Hajdrihova 19, 1000 Ljubljana, Slovenia

[c] Johnson Matthey Technology Centre, Blount's Court, Sonning Common, Reading RG4 9NH, U.K.

[d] Johnson Matthey Fuel Cells, Lydiard Fields, Great Western Way, Swindon SN5 8AT, U.K.

[e] Department of Analytical Chemistry, National Institute of Chemistry, Hajdrihova 19, 1000 Ljubljana, Slovenia

[f] University of Nova Gorica, 5000 Nova Gorica, Slovenia

* to whom correspondence should be addressed: Alex.Martinez.Bonastre@matthey.com, nejc.hodnik@ki.si.

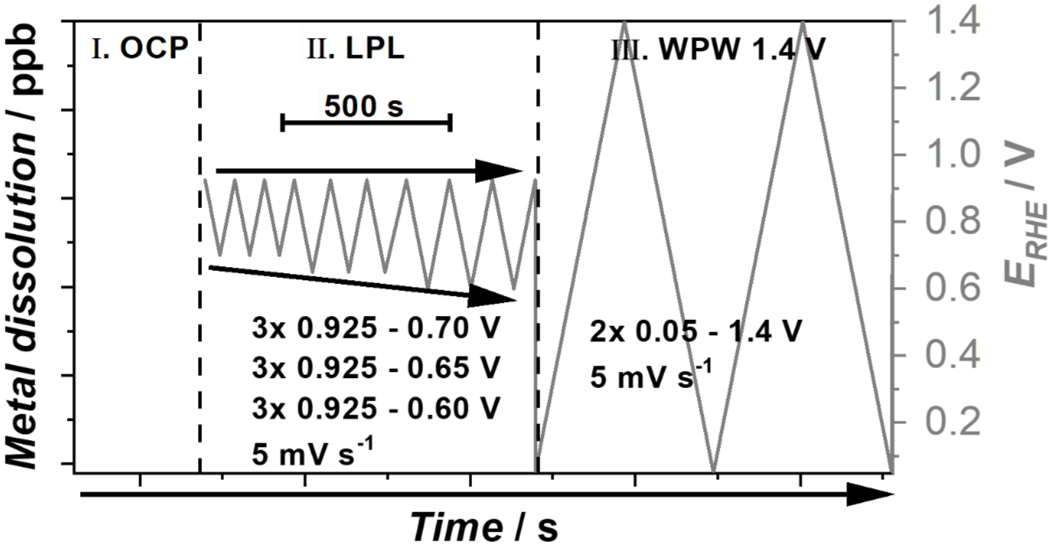

Figure S1. Electrochemical protocol used for evaluation of metal dissolution using EFC-ICP-MS for all electrocatalysts used in this study. The protocol consisted of I. exposing the electrocatalyst to **I. OCP** conditions, followed by **II. LPL** experiment (3 cycles each LPL, 0.925-0.X V_{RHE}; X=70/65/60, 5 mV s^{-1}) and finished by **II. WPW** cycles (0.05-1.4 V_{RHE}, 5 mV s^{-1}, 0.1 M $HClO_4$). All experiments were performed in a flow of 0.1 M $HClO_4$. In addition to the reproducibility, d-Pt-Ni/C-JM was also evaluated for the ‘memory-effect’ at II. LPL part of the experiment by skipping LPLs of 0.7 and 0.65 V by going directly to 0.6 V. Higher dissolution of Ni was observed in the first cycle in contrast to LPL of 0.7 V (**Figure S12**).

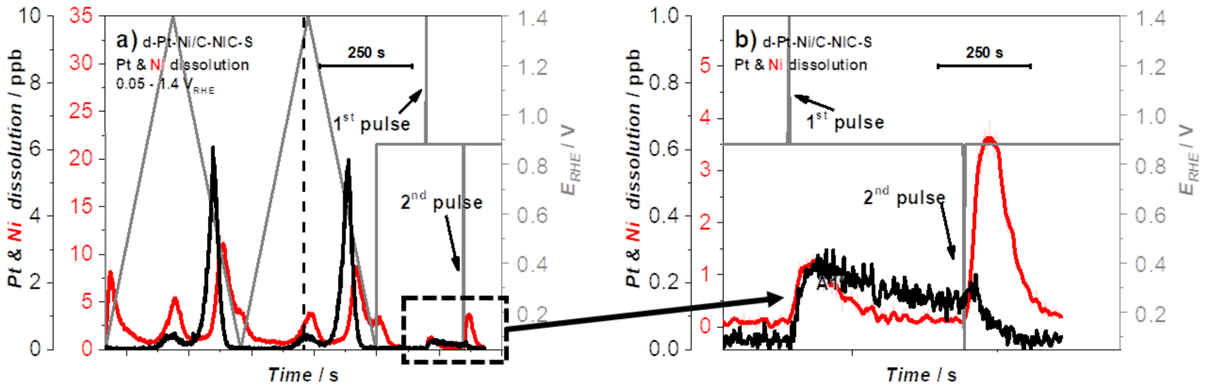

Figure S2. Electrochemical 'pulses' methodology used for synchronization of the electrochemical and ICP-MS signals as exemplified on the d-Pt-Ni/C-NIC-S electrocatalyst.

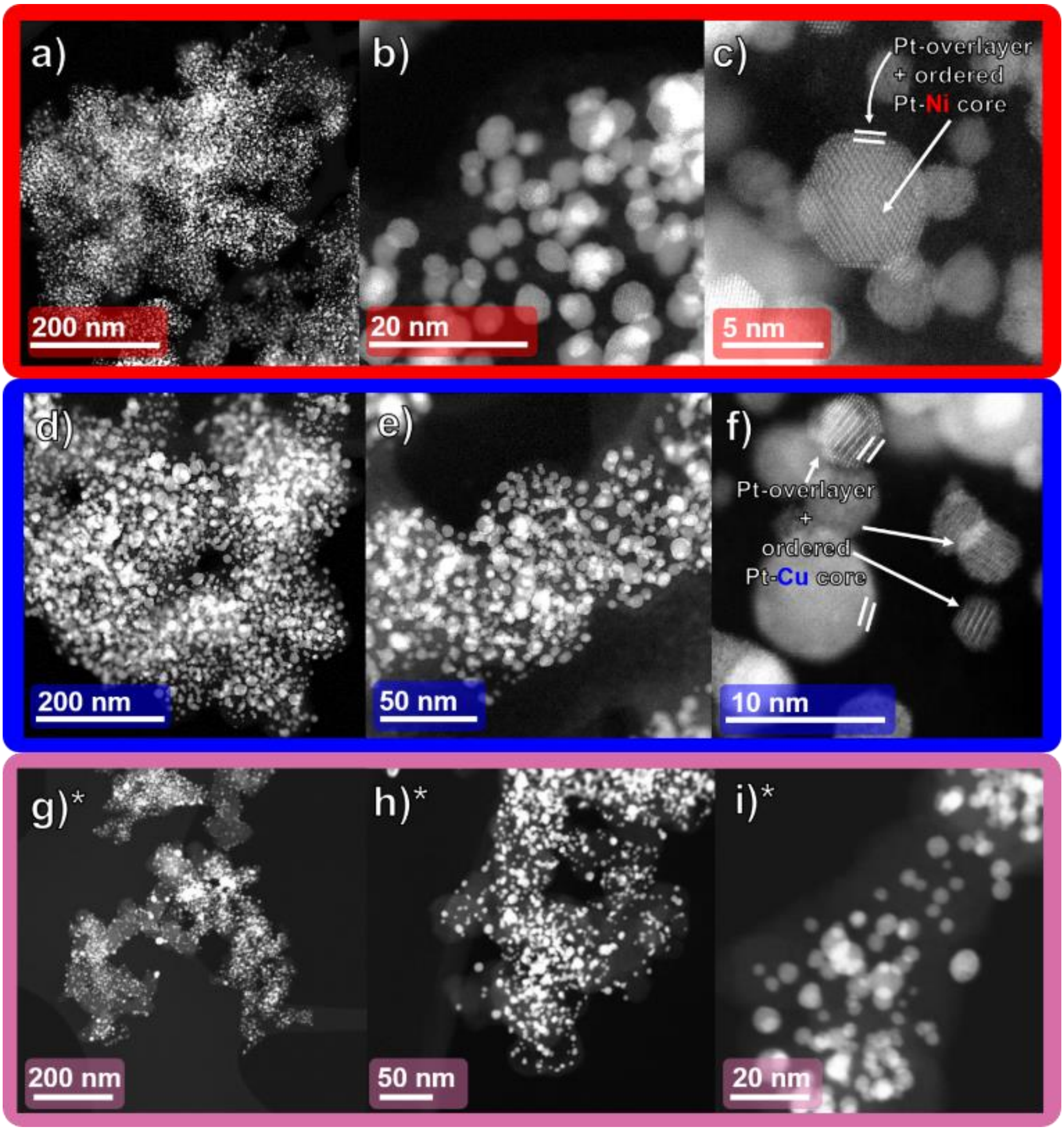

Figure S3. HAADF images of **(a-c)** d-Pt-Ni/C-NIC, **(d-f)** d-Pt-Cu/C-NIC and **(g-i*)** d-Pt-Ni/C-JM electrocatalysts.*taken at Johnson Matthey TEM facilities.

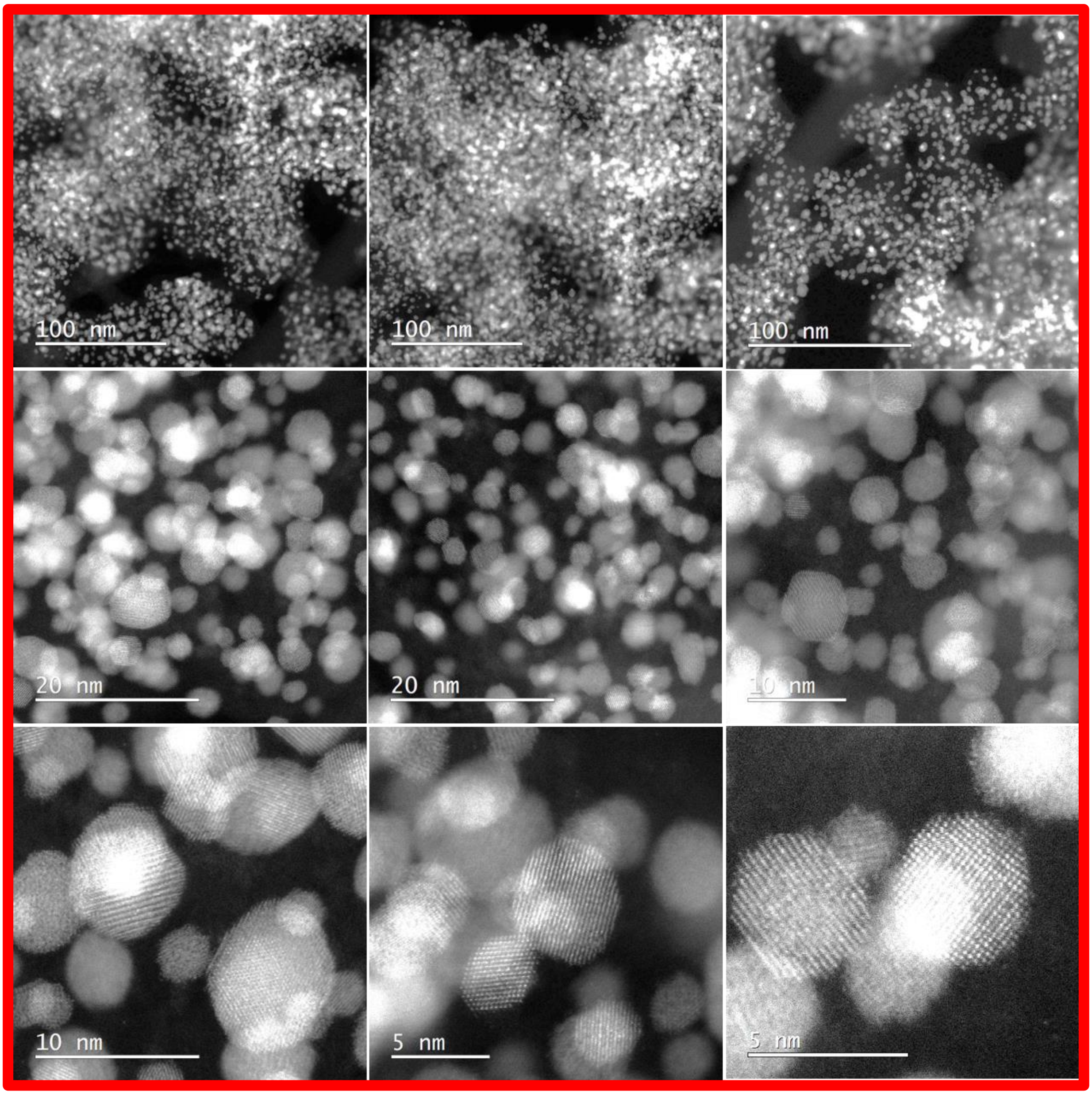

Figure S4. Additional HAADF images of d-Pt-Ni/C-NIC.

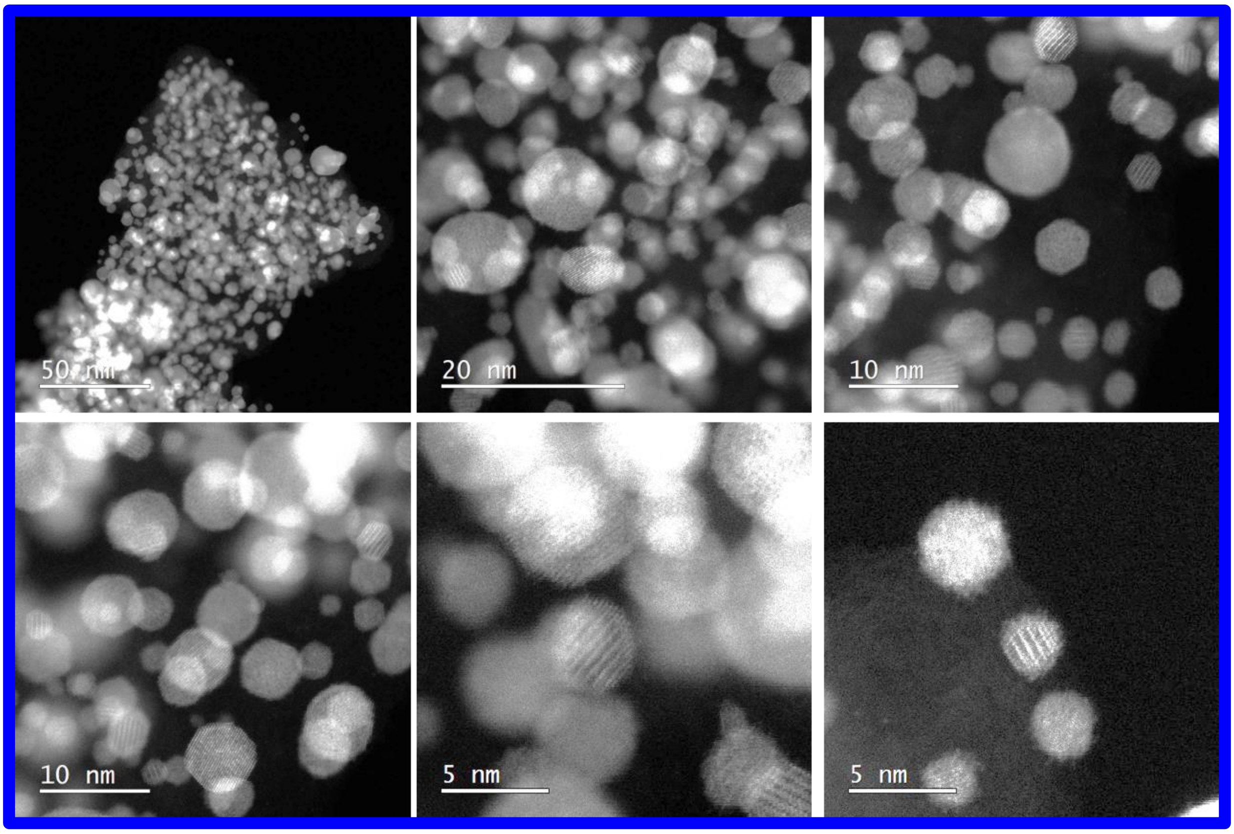

Figure S5. Additional HAADF images of d-Pt-Cu/C-NIC.

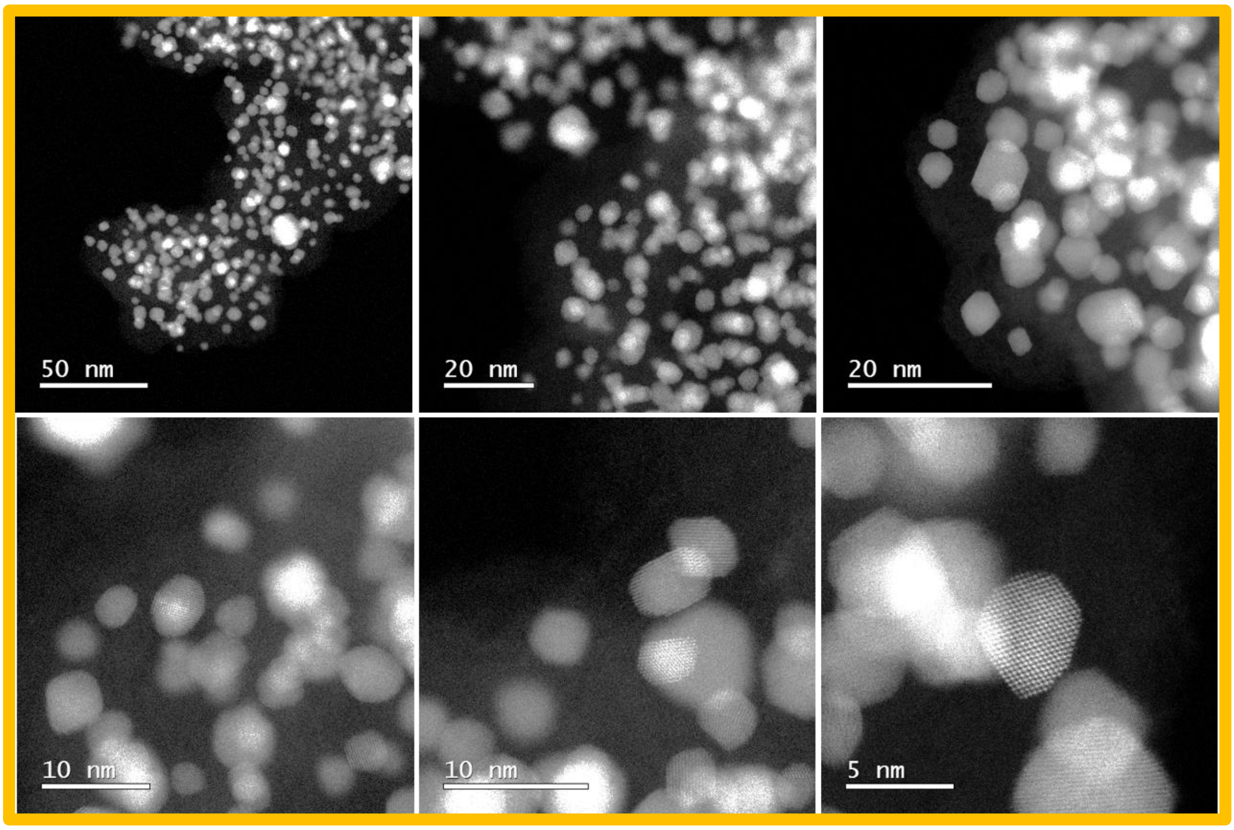

Figure S6. Additional HAADF images of Pt/C (Tanaka Kikinzoku Kogyo, TEC10E50E-HT).

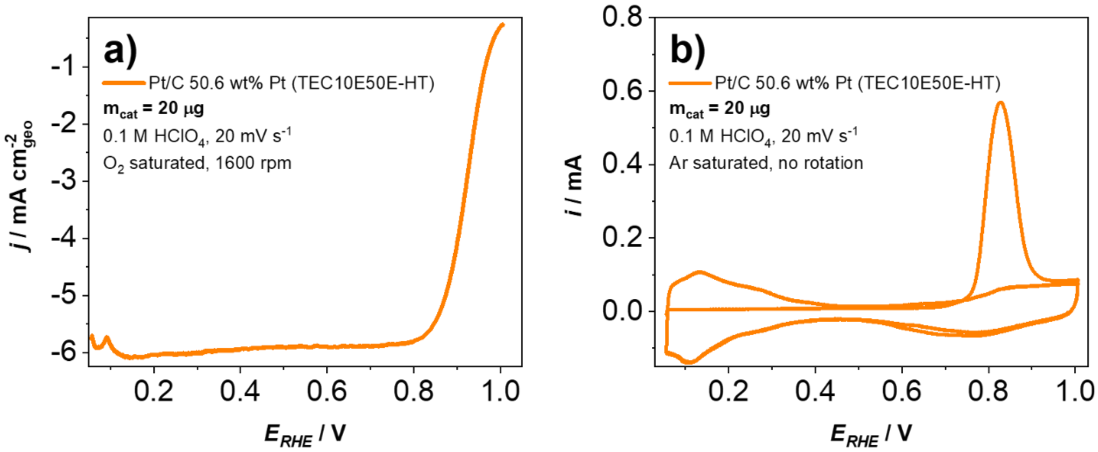

Figure S7. TF-RDE characterization in liquid electrolyte for Pt/C reference (Tanaka Kikinzoku Kogyo, TEC10E50E-HT). **(a)** ORR polarization curve measurement (0.1 M $HClO_4$, 0.05-1.0 V_{RHE}, 20 mV s^{-1}, 1600 rpm, O_2 saturated, ohmic resistance compensated, background corrected) after 200 cycles of PCA (0.1 M $HClO_4$, 0.05-1.2 V_{RHE}, 300 mV s^{-1}, 600 rpm rotation during activation and exchange of electrolyte prior to ORR polarization curve measurements). **(b)** CO electrooxidation experiments (0.1 M $HClO_4$, 0.05-1.0 V_{RHE}, 20 mV s^{-1}, Ar saturated) with their respective follow-up cycles.

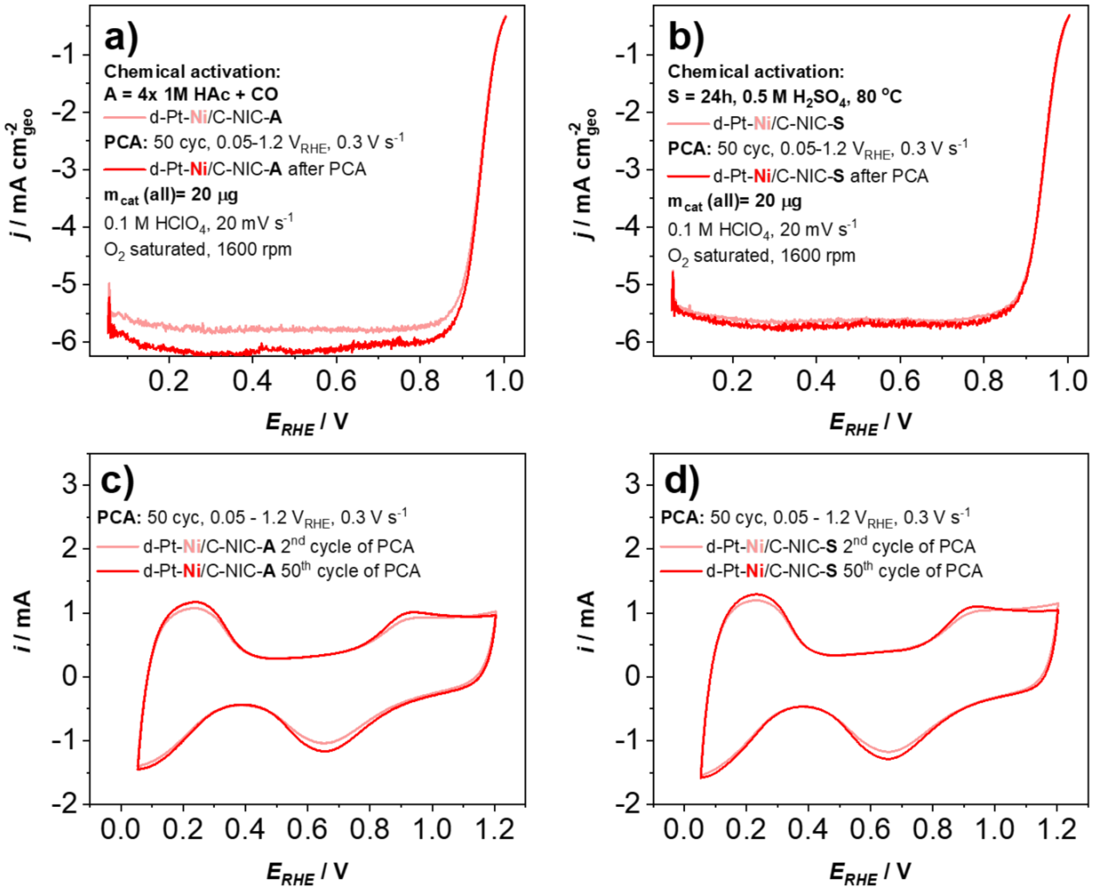

Figure S8. (a-b) Comparison of ORR polarization curves (liquid electrolyte TF-RDE) for d-Pt-Ni/C-NIC-A and d-Pt-Ni/C-NIC-S analogues (0.1 M $HClO_4$, 0.05-1.0 V_{RHE}, 20 mV s^{-1}, 1600 rpm, O_2 saturated, ohmic resistance compensated, background corrected) before and after 50 cycles of PCA (0.1 M $HClO_4$, 0.05-1.2 V_{RHE}, 300 mV s^{-1}, 600 rpm rotation during activation and exchange of electrolyte prior to ORR polarization curve measurements). **(c-d)** Comparison of 2nd and 50th CV of PCA for both d-Pt-Ni/C-NIC-A and d-Pt-Ni/C-NIC-S analogues.

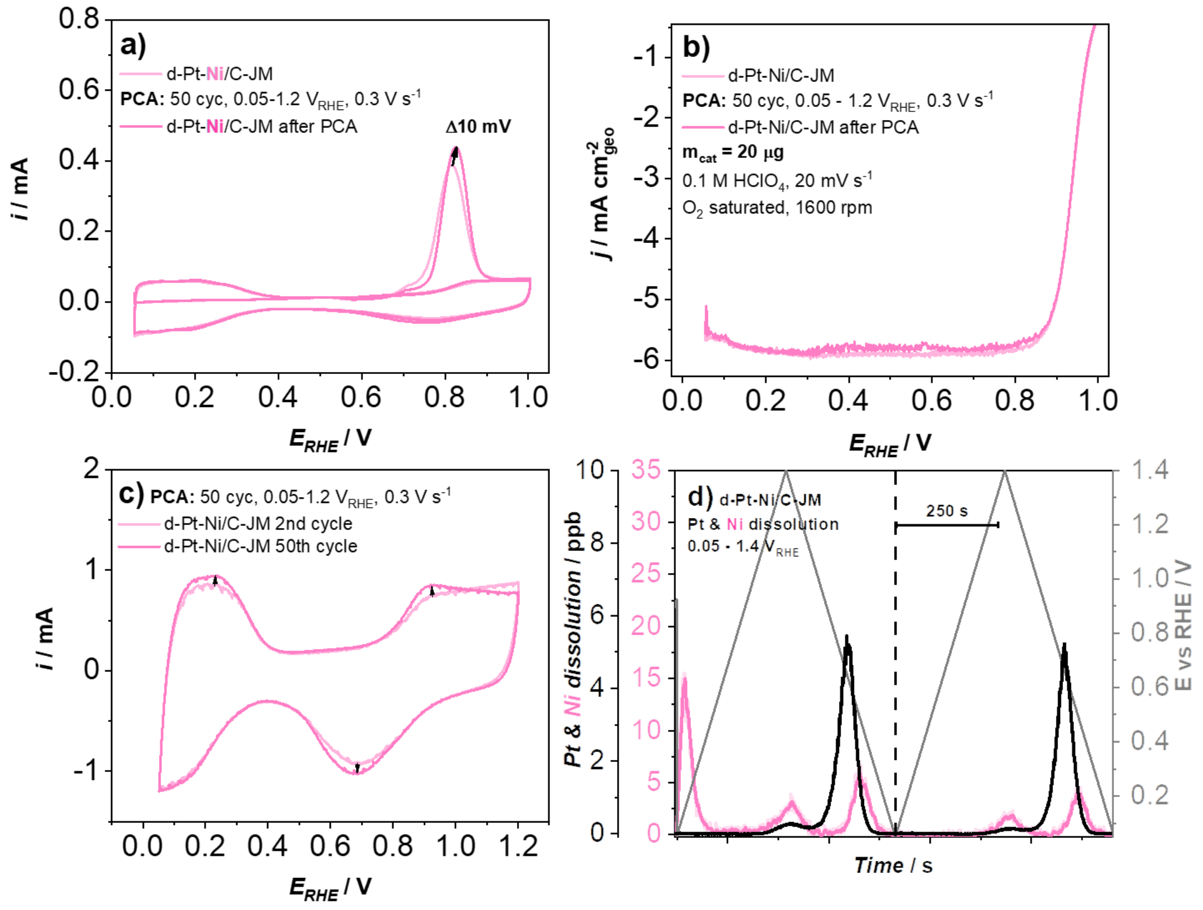

Figure S9. Liquid electrolyte TF-RDE comparison of **(a)** CO electrooxidation experiments (0.1 M $HClO_4$, 0.05-1.0 V_{RHE}, 20 mV s^{-1}, Ar saturated) with their respective follow-up cycles and **(b)** ORR polarization curves for SoA d-Pt-Ni/C-JM benchmark (0.1 M $HClO_4$, 0.05-1.0 V_{RHE}, 20 mV s^{-1}, 1600 rpm, O_2 saturated, ohmic resistance compensated, background corrected) before and after 50 cycles of PCA (0.1 M $HClO_4$, 0.05-1.2 V_{RHE}, 300 mV s^{-1}, 600 rpm rotation during activation and exchange of electrolyte prior to ORR polarization curve measurements). **(c)** Comparison of 2nd and 50th CV of PCA for d-Pt-Ni/C-NIC-JM. **(d)** Pt and Ni dissolution from d-Pt-Ni/C-JM under wide potential window cycles (III. WPW; two cycles 0.05-1.4 V_{RHE}, 5 mV s^{-1}; **Figure S1**).

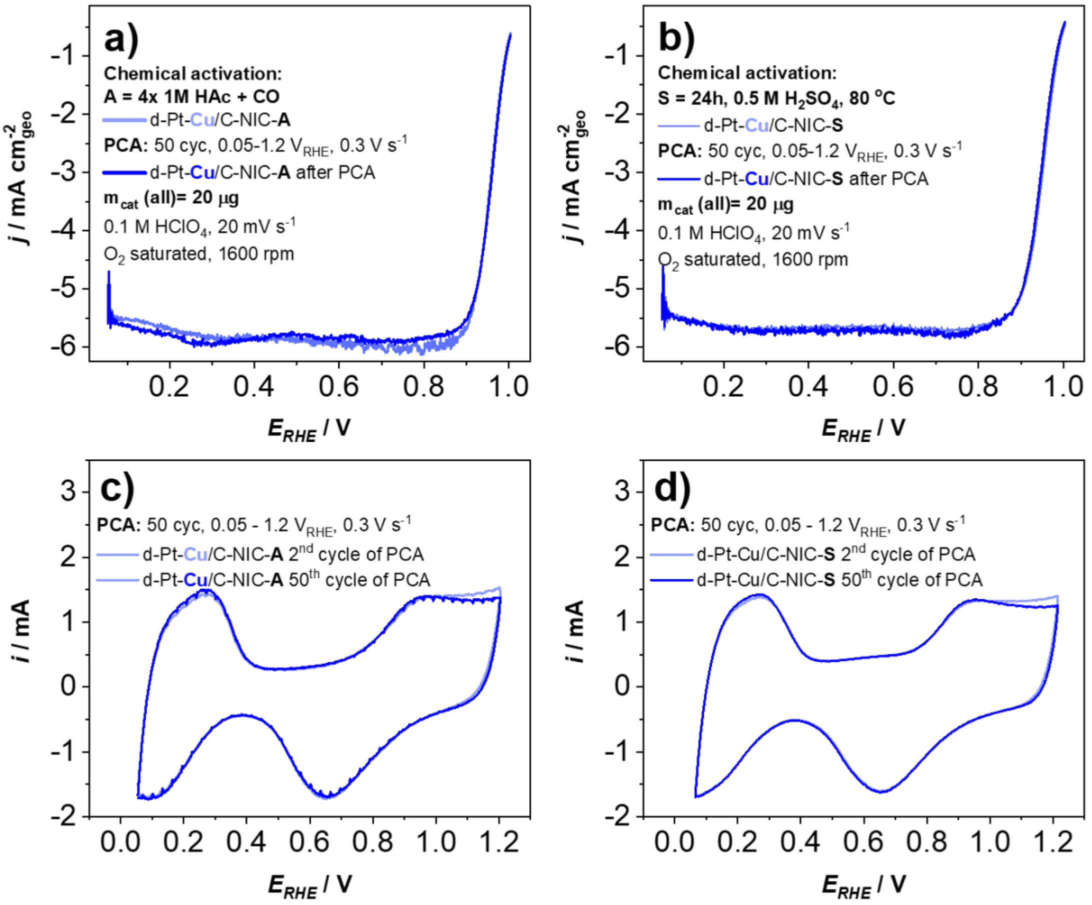

Figure S10. (a-b) Comparison of ORR polarization curves (liquid electrolyte TF-RDE) for d-Pt-Cu/C-NIC-A and d-Pt-Cu/C-NIC-S analogues (0.1 M $HClO_4$, 0.05-1.0 V_{RHE}, 20 mV s^{-1}, 1600 rpm, O_2 saturated, ohmic resistance compensated) before and after 50 cycles of PCA (0.1 M $HClO_4$, 0.05-1.2 V_{RHE}, 300 mV s^{-1}, 600 rpm rotation during activation and exchange of electrolyte prior to ORR polarization curve measurements). **(c-d)** Comparison of 2nd and 50th CV of PCA for both d-Pt-Cu/C-NIC-A and d-Pt-Cu/C-NIC-S analogues.

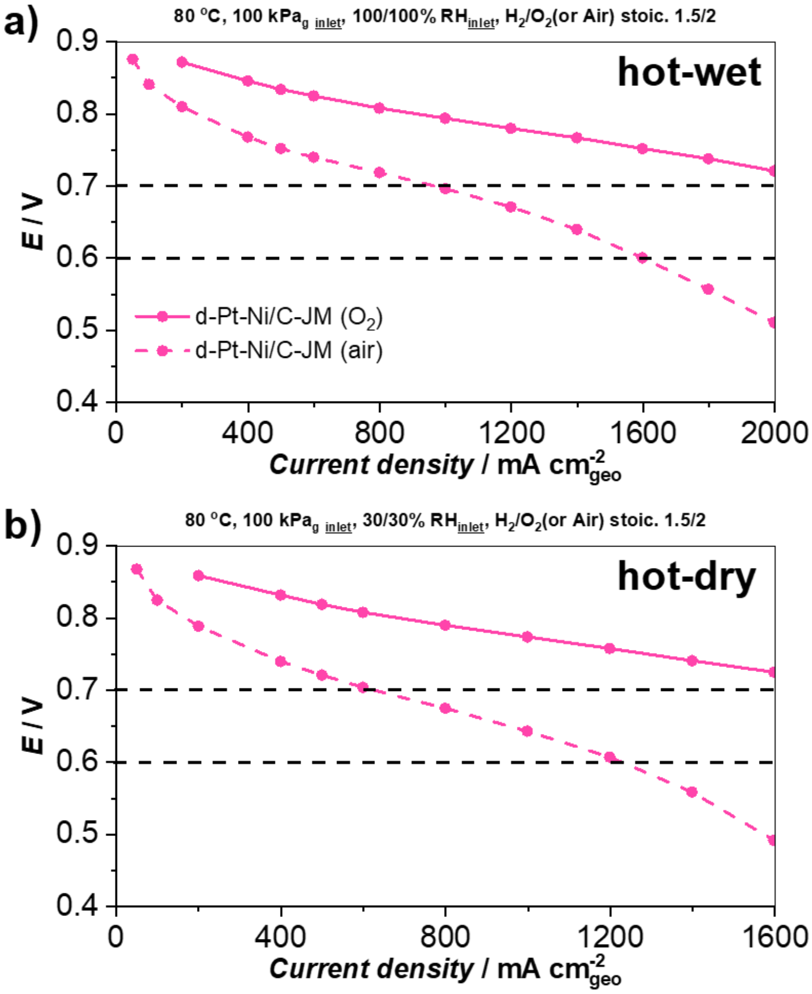

Figure S11. Comparison of ORR polarization curves in a 50 cm^2 single-cell under H_2/O_2 and H_2/Air (anode/cathode) using SoA d-Pt-Ni/C-JM as the cathode catalyst (I/C, loading, kinetic performance and $ECSA_{CO}$) shown in **Table 2** of the main manuscript**)**. General test conditions are specified in both figures. Comparison of SoA d-Pt-Ni/C-JM benchmark at **(a)** hot-wet conditions (80 ºC, 100% RH), **(b)** at hot-dry conditions (80 ºC, 30% RH).

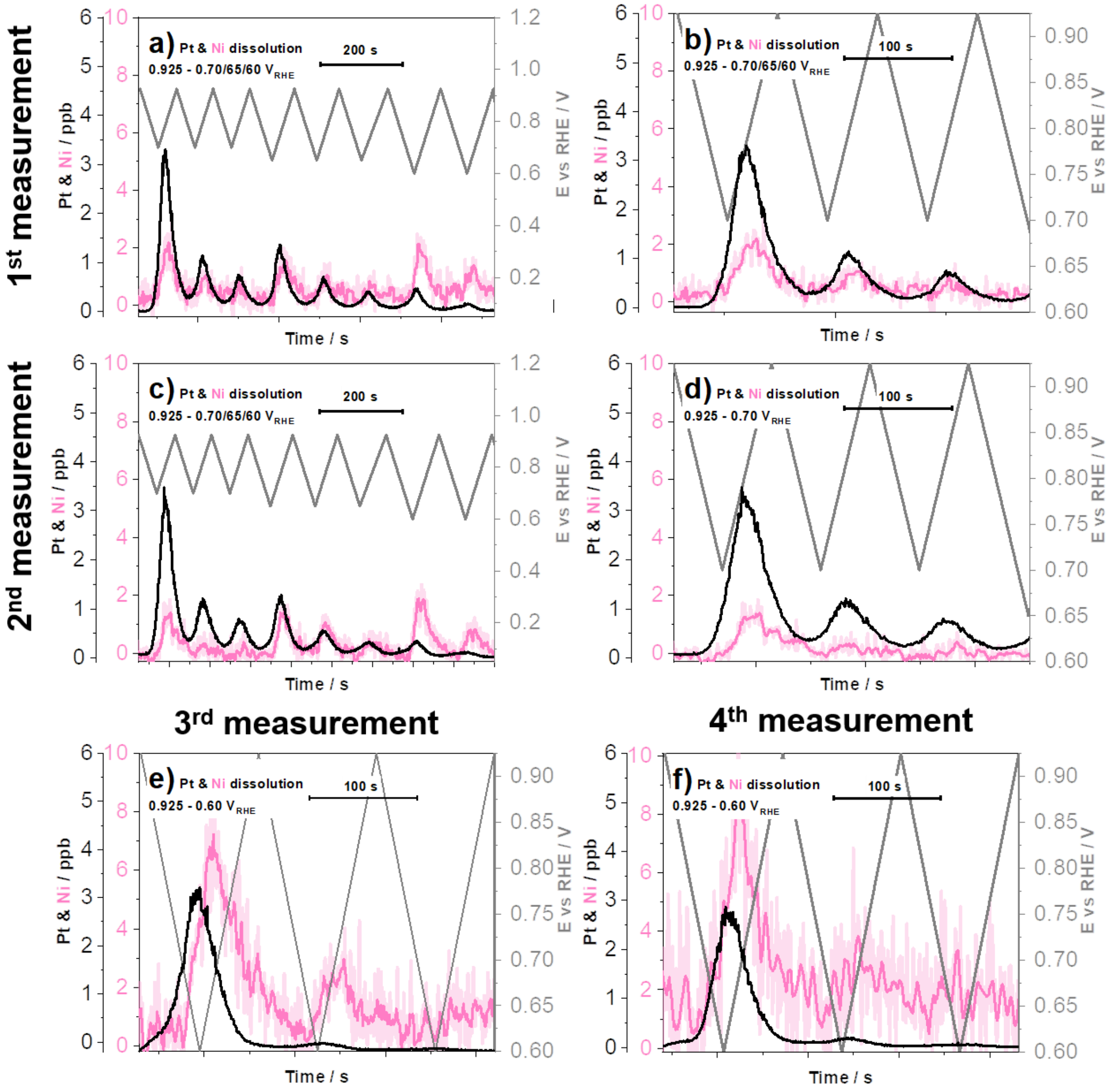

Figure S12. Reproducibility and 'memory-effect' evaluation of the LPL effect using SoA d-Pt-Ni/C-JM benchmark using EFC-ICP-MS in the flow of 0.1 M $HClO_4$. **(a-b)** 1st and **(c-d)** 2nd measurements show reproducibility of the **II. LPL** experiment (3 cycles each LPL, 0.925-0.X V_{RHE}; X=70/65/60, 5 mV s^{-1}). **(e)** 3rd and **(f)** 4th measurement show the 'memory effect' by skipping the LPLs of 0.7 and 0.65 V and going directly to LPL of 0.6 V in the first cycle. By not initiating any prior dissolution, the amount of dissolved Ni in the first cycle far exceeds the amount observed during the **(b)** 1st and **(d)** 2nd measurement, providing further evidence that higher reduction of Pt-oxide leads to a higher degree of cathodic dissolution of Ni already in the operational window.

Table S1. Additional data on the electrocatalysts used in this study such as wt% of Pt and M, mass of Pt and electrocatalyst used for the liquid electrolyte TF-RDE measurements and corresponding area of the CO-electrooxidation peak.

Electrocatalyst	wt% (Pt)	wt% (M)	m_{Pt} [µg]	m_{cat} [µg]	CO-stripping area [cm^2]
**Pt/C TEC10E50E-HT**	50.6	/	10.12	20	5.4
**d-Pt-Cu/C-NIC-A**	36	11.7	7.2	20	5.8
**d-Pt-Cu/C-NIC-S**	33.46	4.79	6.69	20	5.4
**d-Pt-Ni/C-NIC-A**	32	8.35	6.4	20	4.4
**d-Pt-Ni/C-NIC-S**	30.6	4.96	6.12	20	5.0
**SoA d-Pt-Ni/C-JM**	27.4	4.74	5.48	20	3.6
